# VCP enhances autophagy-related osteosarcoma progression by recruiting USP2 to inhibit ubiquitination and degradation of FASN

**DOI:** 10.1038/s41419-024-07168-6

**Published:** 2024-11-03

**Authors:** Shijiang Wang, Jiangbo Nie, Haoxin Jiang, Anan Li, Nanshan Zhong, Weilai Tong, Geliang Yao, Alan Jiang, Xinsheng Xie, Yanxin Zhong, Zhiguo Shu, Jiaming Liu, Feng Yang, Zhili Liu

**Affiliations:** 1 Department of Orthopedic Surgery, The First Affiliated Hospital, Jiangxi Medical College, Nanchang University, Nanchang, 330006 People’s Republic of China; 2 Jiangxi Provincial Key Laboratory of Spine and Spinal Cord Diseases, Nanchang, 330006 People’s Republic of China; 3 Medical Innovation Center, The First Affiliated Hospital of Nanchang University, Nanchang, 330006 People’s Republic of China; 4 Basic Medical School of Nanchang University, Nanchang, 330006 People’s Republic of China; 5 Postdoctoral Innovation Practice Base, The First Affiliated Hospital of Nanchang University, Nanchang, 330006 People’s Republic of China

**Keywords:** Post-translational modifications, Bone cancer

## Abstract

Osteosarcoma (OS) is a highly aggressive malignant tumor with a high rate of disability and mortality rates, and dysregulated autophagy is a crucial factor in cancer. However, the molecular mechanisms that regulate autophagy in OS remain unclear. This study aimed to explore key molecules that affect autophagy in OS and their regulatory mechanisms. We found that fatty acid synthase (FASN) was significantly increased in activated autophagy models of OS and promoted OS proliferation in an autophagy-dependent manner, as detected by LC3 double-labeled fluorescence confocal microscopy, western blotting, transmission electron microscopy (TEM), and cell functional experiments. Furthermore, co-immunoprecipitation combined with mass spectrometry (Co-IP/MS), ubiquitination modification, molecular docking, and protein truncation methods were used to identify FASN-interacting proteins and analyze their effects on OS. Valosin-containing protein (VCP) enhanced the FASN stability by recruiting ubiquitin specific peptidase-2 (USP2) to remove the K48-linked ubiquitin chains from FASN; domain 2 of VCP and the amino acid sequence () of USP2 were critical for their interactions. Gain- and loss-of-function experiments showed that the inhibition of FASN or USP2 attenuated the stimulatory effect of VCP overexpression on autophagy and the malignant phenotypes of OS cells in vitro and in vivo. Notably, micro-CT indicated that VCP induced severe bone destruction in nude mice, which was abrogated by FASN or USP2 downregulation. In summary, VCP recruits USP2 to stabilize FASN by deubiquitylation, thereby activating autophagy and promoting OS progression. The identification of the VCP/USP2/FASN axis, which mediates autophagy regulation, provides important insights into the underlying mechanisms of OS and offers potential diagnostic and therapeutic strategies for patients with OS.

## Introduction

Osteosarcoma (OS) is the most common malignant bone tumor in children and adolescents. It commonly occurs in the middle of the long bones of the lower limbs and occasionally in the upper limbs and pelvis [1, 2]. Early and accurate diagnosis, preoperative chemotherapy, surgery, and postoperative chemotherapy are critical for this complex and potentially fatal disease [3]. Nonetheless, the survival rate of patients with OS remains relatively low owing to the complex biological origin of the disease and the poorly understood mechanisms underlying recurrence and lung metastasis [4, 5]. Therefore, understanding the molecular mechanisms that drive the proliferation and metastasis of OS cells and identifying potential molecular targets are critical steps in the effective development of antitumor drugs.

Autophagy is universally recognized as a degradation and recycling system triggered by the release of nutrients, which maintains cellular homeostasis by degrading redundant or dysfunctional proteins and organelles [6, 7]. Moreover, autophagy facilitates the propagation and proliferation of tumors by providing nutrients and energy to the microenvironment created by tumor cells [8]. The activity of autophagy varies among different cancers, including colon, lung, and breast cancers [9]. The activation of autophagy promotes OS progression and is highly correlated with disease prognosis. For example, autophagy-related genes BECN1 and LC3B-II are significantly overexpressed and highly correlated with the malignant progression of OS [10]. However, the intricate regulatory network governing autophagy activation in OS cells remains obscure, necessitating further elucidation to fully comprehend its role in OS development and progression.

Fatty acid synthase (FASN) is a crucial enzyme involved in lipid metabolism that catalyzes the final steps of de novo fatty acids [11]. Upregulation of FASN is associated with the progression of several cancers, including prostate [12], ovarian [13], breast [14], liver [15] and cervical cancers [16]. Furthermore, we found that FASN was differentially expressed between normal and OS tissues, while promoting OS cell proliferation and metastasis [17, 18]. Additionally, long-chain fatty acids synthesized by FASN catalysis are a rich source of lipids for the formation of autophagosomes and autophagic lysosomes [19]. Autophagy and FASN levels were also elevated in HCC and correlated with disease prognosis [20]. Nevertheless, the effects of FASN proteins on OS autophagy and malignant behavior remain unclear.

In this study, we identified FASN as a key factor contributing to autophagy in OS and found that elevated FASN expression was associated with a nutrient-deprived microenvironment, tumor malignancy, and autophagy levels. Mechanistically, nutrient deficiency strengthens the interaction between FASN and VCP and recruits ubiquitin specific peptidase-2 (USP2) to reprogram FASN stability, ultimately promoting autophagy and progression of OS cells. Our study identified a novel autophagy regulator in OS, which may provide clues for therapeutic intervention in patients with OS.

## Materials and methods

### Cell culture

The 293 T and U2OS cells were obtained from ProCell (Danvers, MA, USA). The 143B cells were purchased from the American Type Culture Collection (ATCC, USA). All these cell types were cultured in McCoy’s 5 A and Dulbecco’s Modified Eagles Medium. All cells were cultured in a complete medium supplemented with 10% FBS (Gibco, USA) and 1% antibiotics (100 units/mL each of penicillin and streptomycin) in a humidified incubator at 37 °C and 5% CO^2^ atmosphere. All cells were identified by STR profiling.

### Real-time quantitative PCR (RT-qPCR)

The quantitative real-time PCR (qRT-PCR) assays were performed as described previously [21, 22]. Total RNA was extracted using Total RNA Extraction Reagent (#EZB-TZ1, EZBioscience, USA) and reverse-transcribed using the HiScript II 1st Strand cDNA Synthesis Kit (#R211-01, Vazyme, China). qRT-PCR was then performed using Hieff UNICON® Universal Blue qPCR SYBR Green Master Mix (#11184ES08, YEASEN, China). The primer sets used are listed in Supplementary Table S5. ACTB mRNA was used as a control. The 2^-ΔΔCt^ method was used for relative expression analysis. These experiments were repeated three times.

### Western blot

Total cellular proteins were extracted using the RIPA lysis buffer (#89900, Thermo Scientific^TM^, USA). The proteins on the PVDF membranes were incubated with primary antibodies: anti-LC3B (ab63817), anti-FASN (ab128870), anti-VCP (ab109240), anti-USP2 (ab66556, Abcam, USA), anti-SQSTM1/p62 (#23214), anti-Beclin-1 (#3738), anti-HA-tag (#3724), anti-His-Tag(#12698), anti-PCNA (#2586), anti-Bcl-xl (#2764), anti-ACTB (#4970), and anti-MYC-Tag(#2276, CST, USA), anti-FLAG-Tag(#AE005, Abclonal, China).

### CRISPR/dCas9-mediated inhibition and activation

The clustered regularly interspaced short palindromic repeat (CRISPR**/**dCas9) approach was employed [23]. The appropriate single-guide RNAs (sgRNAs) were designed (Supplementary Table S6) and cloned into the Lenti-CRISPR/dCas9-VPR or Lenti-CRISPR/dCas9-KRAB vector according to our previously described method to produce CRISPRi (Ci) and CRISPRa (Ca) constructions [24]. Infectious lentiviruses were produced and harvested from HEK-293T cells as described in a previous study [24]. Cells were infected with lentivirus and cultured with puromycin.

### Cell proliferation assay

An Edu kit (Riobio, USA) and colony formation assays were used to detect OS cell proliferation. Cells were incubated with 50 μM Edu reagent in a confocal petri dish according to the manufacturer’s protocol. Nuclei were stained with Hoechst, and the results were recorded using a confocal microscope (STELLARIS 5, Leica).

For the colony formation assay, 1 × 10^3^ cells per well were plated in 6-well plates and cultured for 14 days. The colonies were fixed with 4% paraformaldehyde for 30 min and stained with 0.1% crystal violet for 15 min. Colonies containing >50 cells were counted using the ImageJ (version 1.8.0.345).

For the CCK-8 assay, the cells were seeded in 96-well plates at a density of 4 × 103 cells per well. Following incubation overnight, the cells were exposed to CB-5083 (MedChem Express, HY-12861) for 24 h. After 24 h of incubation, absorbance at 450 nm was measured using Cell Counting Kit-8 (YESEN, 40203ES76). We then established the cell viability curve and calculated the IC50 value using GraphPad Prism software (Prism 8).

### Cell apoptosis detection assay

An Annexin V-APC/PI double staining kit (#BB-41033, BestBio, China) was used to evaluate cell apoptosis. The cells were digested, suspended, and centrifuged at 300 × *g* for 5 min. After washing with pre-cooled PBS, cells were collected by centrifugation at 300 × *g* for 5 min. Binding buffer (500 μL) was added to resuspend the cells. Next, the cells were incubated with 5 μL Annexin V-FITC at 4 °C for 10 min, immediately followed by staining with 5 μL PI for 5 min. Apoptosis was detected using the NovoCyte Penteon flow cytometer (Agilent Technologies).

### Cell migration and invasion assays

For the migration assay, the upper chambers of the Transwell chamber were seeded with 2 × 10^4^ cells suspended in 200 μL of serum-free medium. Meanwhile, the lower chambers were filled with 800 μL of basal medium containing 20% fetal bovine serum (FBS). The cells were incubated for 24 h, then stained with crystal violet for analysis.

For the invasion assay, the upper surface of the membrane was first coated with 40 μL of a 1:8 Matrigel and culture medium mixture. Afterward, 4 × 10^4^ cells in 200 μL of serum-free medium were placed in the upper chamber, while 800 μL of basal medium with 20% FBS was added to the lower chamber. Following a 24 h incubation at 37 °C, the cells were stained with crystal violet.

### Immunofluorescence (IF) staining

To determine the subcellular distribution of FASN, VCP, and USP2, 1.5 × 10^5^ OS cells were inoculated into glass-bottomed dishes 24 h in advance. On day 2, cells were fixed with 4% paraformaldehyde for 15 min. After washing with PBS three times, the cells were treated with 0.5% Triton X-100 for 15 min. After 1 h of incubation with the appropriate dilution of the primary antibody, the cells were washed three times with PBS and incubated for 1 h with a fluorescent secondary antibody. The cell nuclei were stained with DAPI (#62248, Thermo Scientific) and visualized using a confocal microscope (Leica).

### Analysis of autophagic flux

To monitor autophagic flux, LC3 cells were labeled using RFP-GFP-LC3 lentivirus. The treated OS cells were transfected with RFP-GFP-labeled lentiviruses for 48 h. Images were obtained by confocal microscopy. Yellow dots (combining GFP and RFP signals) represent early autophagosomes, and red dots (RFP signals only) represent late autophagosomes.

### Murine models and tumor imaging

All animal experiments were performed in accordance with the National Institutes of Health Guidelines for the Care and Use of Laboratory Animals and approved by the ethics committee of the First Affiliated Hospital of Nanchang University. A total of 48 four-week-old female nude mice were procured from the Zhejiang Resources Laboratory Animal Center, with 24 female nude mice used for subcutaneous xenograft models and the remainder for orthotopic xenograft experiments. The mice were subsequently randomly divided into four groups, with six mice in each group. For subcutaneous xenograft assays, 2 × 10^6^ transfected OS cells were implanted in the right rear flank of mice. For the orthotopic xenograft experiments, 20 µL of 143B suspension (2 × 10^7^ cells/mL) was injected into the proximal tibia. The body weight and tumor size of each nude mouse were measured every 3 days. The tumor volume was calculated using the formula *V* = 1/2 × L × W^2^ (where L and W are the length and width of the tumor, respectively). Orthotopic xenograft mice were scanned using micro-computed tomography (Mlabs) before sacrifice. Tumor and lung tissues were collected for H&E and IHC staining.

### H&E and IHC staining

Tumor and lung tissues were separated, fixed with 4% paraformaldehyde, dehydrated, and embedded in paraffin wax. Then, paraffin-embedded tissues were cut into 4 millimeter sections using a microtome, flattened in water at 45 °C, and baked at 70 °C for 30 min. Tumor and lung sections were dewaxed and histologically analyzed using H&E staining. Antibodies specific for FASN, VCP, and USP2 were introduced, and incubation was initiated. Finally, the intensities were measured and imaged.

### Statistical analysis

GraphPad Prism 8.0.2 (La Jolla, USA) was used for data analysis, with the resulting data presented as the mean ± standard error of the mean (SEM). The mean difference between groups was analyzed by *T*-test or ANOVA. *P* < 0.05 was considered statistically significant.

## Results

### FASN facilitates autophagy in OS

**Fig. 1:**
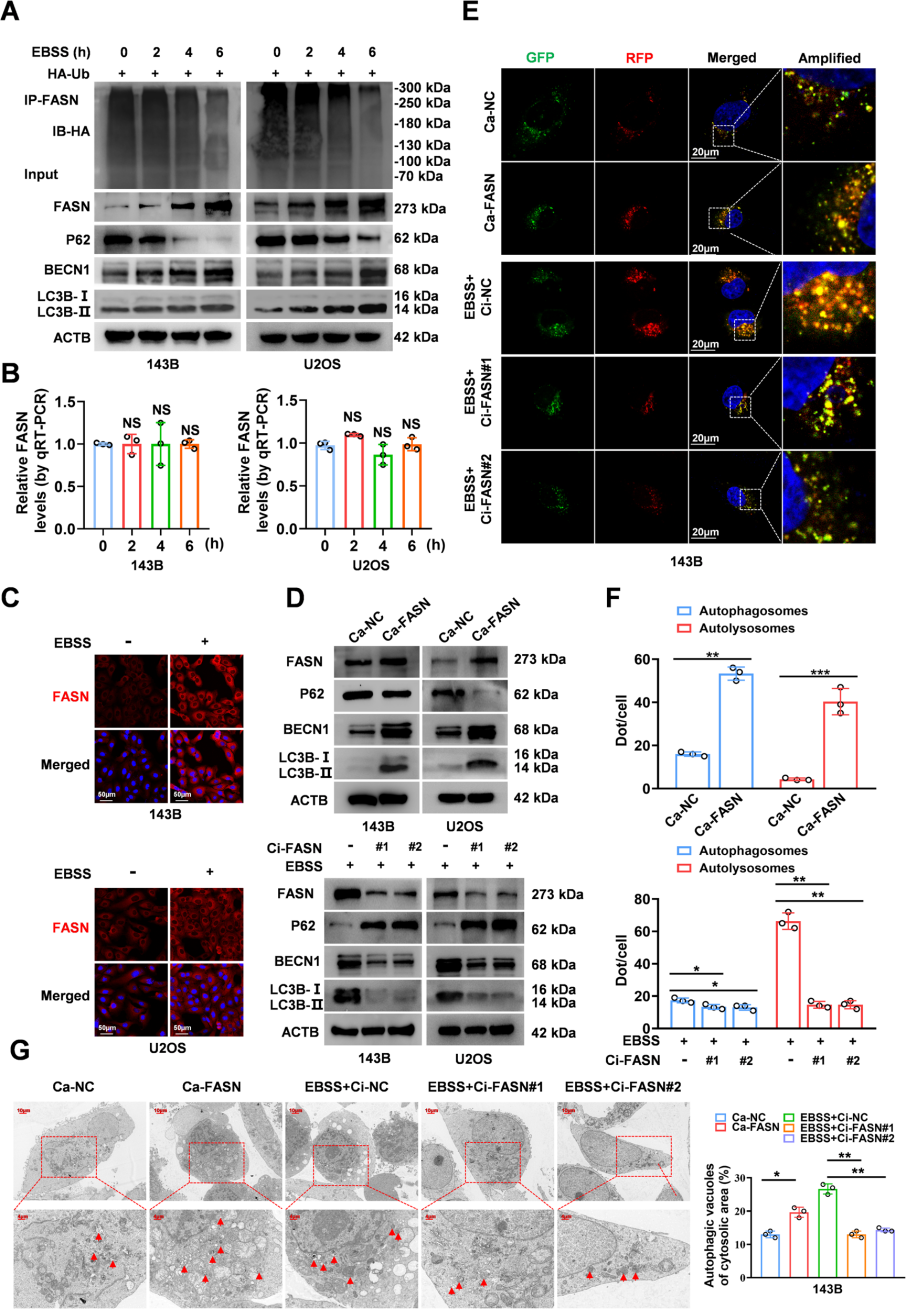
FASN facilitates autophagy in OS. **A** Western blot was conducted to analyze the levels of autophagy-related proteins, FASN, and FASN ubiquitination in 143B and U2OS cells treated with EBSS for 0, 2, 4, and 6 h. **B** Real-time qRT-PCR analysis showed that the level of FASN mRNA changed after EBSS treatment. **C** Immunofluorescence analysis was utilized in the upper figure to compare the fluorescence intensity of FASN after treatment with EBSS (-) and EBSS (+) for 6 h in 143B and U2OS cells; quantification is shown below. **D** Western blotting displaying the expression of autophagy-associated proteins. **E**, **F** Representative images and quantitative results revealing the intensity of RFP-GFP-LC3 immunofluorescence staining in 143B cells stably transfected with Ca-FASN and treated with CiFASN#1 or Ci-FASN#2 then induced with EBSS. Red dots represent autolysosomes and yellow dots represent autophagosomes. Scale bar: 20 µm. **G** TEM scanning displaying autophagic vacuoles in 143B cells treated with Ca-FASN and treated with CiFASN#1 or Ci-FASN#2 then induced with EBSS. *P < 0.05.

We observed that LC3B and BECN1 significantly increased under Earle’s balanced salt solution (EBSS) starvation conditions, with a decrease of P62 protein, importantly accompanied by a dramatic increase in the levels of FASN protein but not mRNA levels. Moreover, there was also a significant decrease in the ubiquitination level of FASN (Fig. 1A, B). In addition, immunofluorescence (IF) assays revealed that the fluorescence intensity of FASN was consistently elevated in EBSS-treated OS cells (Fig. 1C). These results indicate that FASN protein levels were significantly upregulated during autophagy activation, suggesting that FASN may be involved in the autophagy activation process in OS. As expected, FASN overexpression activated autophagy, whereas FASN deletion effectively inhibited EBSS-mediated autophagy in OS cells (Fig. 1D). To dynamically observe the process of autophagic flow, we transfected the RFP + GFP LC3 lentiviral plasmid into OS cells. Confocal and transmission electron microscopy (TEM) results indicated that FASN overexpression accelerated autophagic flow and promoted the formation of autophagic vesicles in OS cells, whereas its downregulation hindered the activation of autophagy (Fig. 1E–G and Supplementary Fig. S1A, B). Taken together, these results suggest that FASN plays a vital role in regulating autophagy in OS cells.

### FASN promotes OS tumorigenesis in an autophagy-dependent manner

**Fig. 2:**
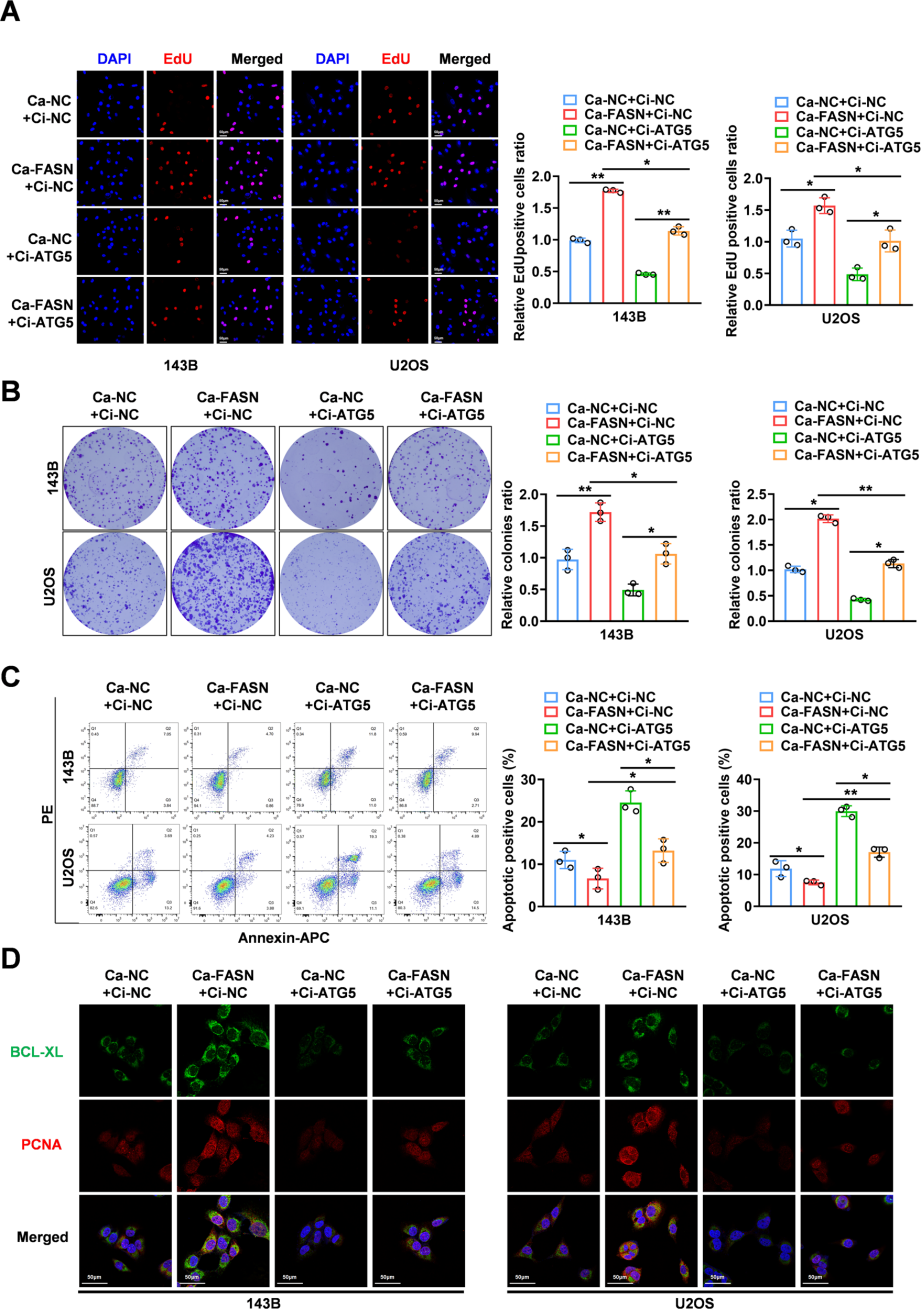
FASN promotes cancer progression through autophagy enhancement. **A**–**C** Representative images (left) and the quantification (right) of Edu, colony formation and flow cytometry assay showing the growth and apoptotic rate of 143B and U2OS cells stably transfected with Ci-ATG5 or Ca-FASN or co-transfected with Ci-ATG5. **D** Immunofluorescence assay for the detection of the fluorescence intensity of cell proliferation protein (PCNA) and cellular anti-apoptotic protein (Bcl-xl) in 143B and U2OS cells stably transfected with Ci-ATG5 or Ca-FASN or co-transfected with Ci-ATG5. Scale bar: 50 µm *P < 0.05.

To further verify the role of FASN in regulating cellular autophagy in the biological function of OS, we constructed rescue models of autophagy activation and inhibition in OS cells via the overexpression or downregulation of ATG5 (autophagy-related 5) [25, 26]. The Edu and colony formation assays confirmed that ATG5 downregulation significantly inhibited the proliferative ability of 143B and U2OS cells overexpressing FASN (Fig. 2A–B, and Supplementary Fig. S2A, B). Flow cytometry and Bcl-xL protein analyses were used to quantify apoptosis. We found that the apoptotic ratio was elevated when FASN was not present and decreased in the presence of FASN, whereas downregulation or overexpression of ATG5 inhibited the regulatory effect of FASN in OS cells (Fig. 2C, D and Supplementary Fig. S2C, D). To clarify whether FASN promotes OS cell migration, invasion, and chemoresistance in an autophagy-dependent manner, we established an ATG5 knockdown rescue model. Transwell assays demonstrated that ATG5 downregulation significantly inhibited the effect of migration (Supplementary Fig. S3A) and invasion (Supplementary Fig. S3B) in OS cells induced by FASN. Cell viability was assessed after 24 h treatment with different concentrations of cisplatin, and the results indicated that FASN overexpression reduced cisplatin sensitivity, while ATG5 knockdown attenuated this effect (Supplementary Fig. S3C). In conclusion, FASN not only promotes the proliferative capacity of OS cells in an autophagy-dependent manner but also plays a critical role in invasion, migration, and resistance to chemotherapy.

### VCP cooperates with FASN to facilitate its expression

**Fig. 3:**
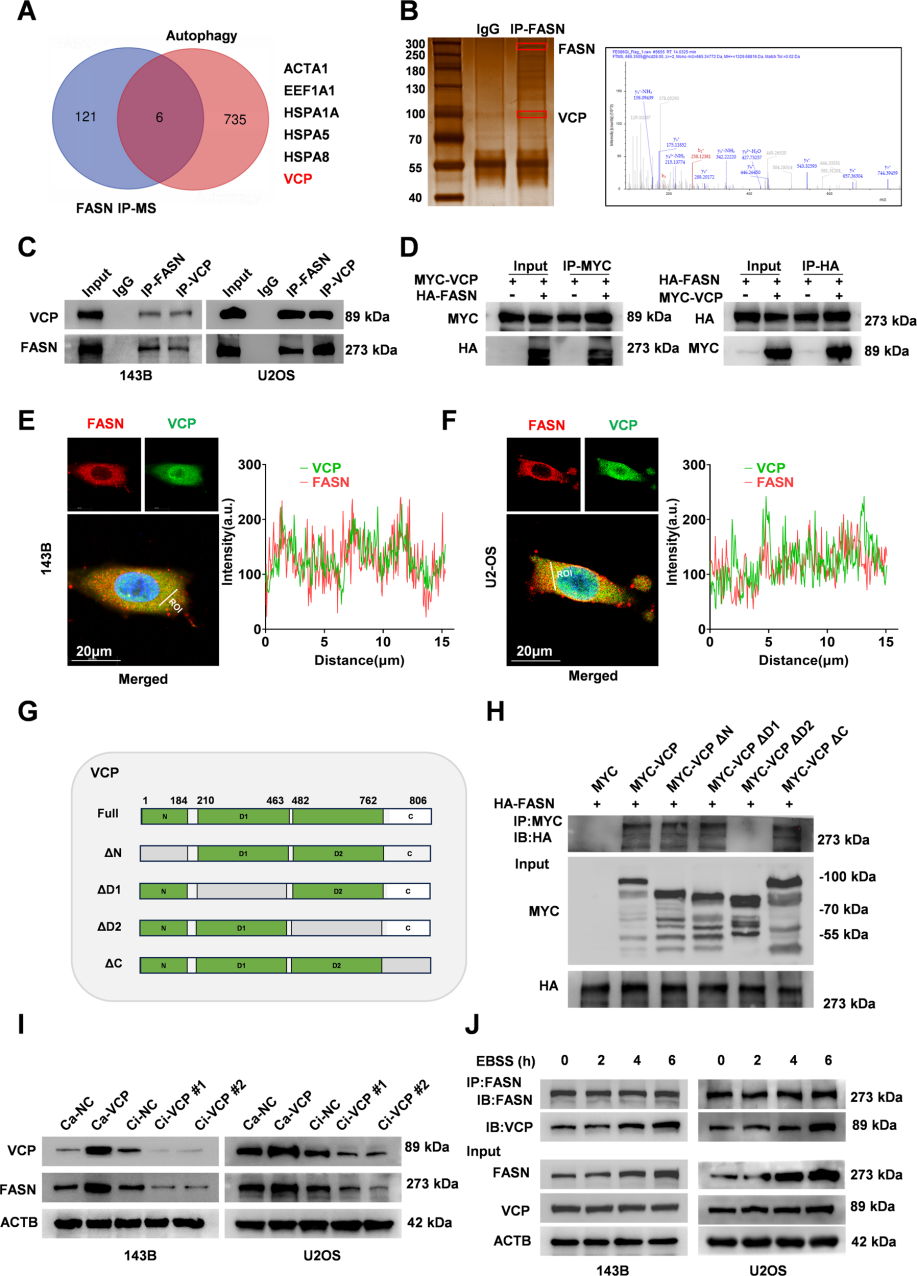
VCP cooperates with FASN to facilitate its expression. **A** Overlapped results combining autophagy database and FASN mass spectrometry analyses. **B** Silver staining assay(left) to detect proteins cooperating with FASN proteins and VCP peaks in protein mass spectrometry(right). **C**, **D** Validation of VCP-FASN interaction in 143B, U2OS and 293 T cells using CoIP assay. **E**, **F** Representative images (left) and the quantification (right) of immunofluorescence demonstrate the localization of FASN and VCP proteins in 143B and U2OS cells. **G** VCP truncated structure. The wild-type VCP, consisting of 806 amino acids, is divided into four structural domains: ΔN, ΔD1, ΔD2, and ΔC tail. Gray areas indicate deleted regions. **H** 293 T cells were transfected with MYC-labeled full-length VCP and its mutants with deletions in each region. Cell lysates were immunoprecipitated with anti-MYC antibody. Western blot assay was conducted to identify the expression of the MYC probe and FASN. **I** Western blot assay was performed to identify alterations in FASN protein levels resulting from stable transfection of Ca-NC, Ca-VCP, Ci-NC, Ci-VCP#1, or Ci-VCP#2. **J** CO-IP assay was utilized to measure the potency of FASN interactions with VCP, following treatment with EBSS for durations of 0, 2, 4, and 6 h.

The protein level of FASN, rather than its mRNA level, significantly increased during autophagy activation in OS cells. Notably, FASN ubiquitination was inhibited during autophagy activation. Therefore, we hypothesized that deubiquitylation of FASN may play an important role in autophagy activation. To elucidate the regulatory mechanisms of FASN deubiquitylation during autophagy activation, we conducted co-immunoprecipitation (co-IP) and mass spectrometry to determine the essential protein associated with FASN in OS and identified 127 proteins (Supplementary Table S1). Subsequently, a list of cellular autophagy-related proteins was obtained from the Autophagy Database (http://www.tanpaku.org/autophagy/) (Supplementary Table S2). Overlapping analysis revealed that six autophagy-regulated proteins may be involved in the regulatory role of FASN (Fig. 3A). Among them, VCP promotes OS proliferation and metastasis via autophagy induction pathway in tumors cells [27–29], and VCP could be an important part of the ubiquitin proteasome system [30]. we further investigated the effect of VCP on autophagy in OS cells and found that VCP promoted autophagy (Supplementary Fig. S4A–C). To clarify whether the stability of FASN is regulated by VCP, we performed Co-IP and IF experiments, based on the results of which, we concluded that VCP and FASN reciprocally precipitated in 143B, U2OS, and 293 T cells and predominantly found in the cytoplasm of OS cells (Fig. 3B–F). To elucidate the key structural domains that are responsible for the interaction between FASN and VCP, a series of FASN truncation mutants (full-long, ΔN, ΔD1, ΔD2, ΔC tail) was constructed based on the protein structure [31, 32] (Fig. 3G). The results of Co-IP assays revealed that deletion of the D2 structural domain in the VCP significantly disrupted its interaction with FASN (Fig. 3H). To further elucidate the regulatory relationship between VCP and FASN, we constructed stable cell lines overexpressing or downregulating VCP. VCP promoted the protein expression of FASN rather than its mRNA expression (Fig. 3I and Supplementary Fig. S5A). Importantly, the interaction between VCP and FASN significantly increased following EBSS treatment (Fig. 3J). Collectively, VCP cooperates with FASN to facilitate its expression, and importantly, starvation stimulation increases protein interactions.

### VCP promotes FASN stability by inhibiting ubiquitination modification of FASN proteins

**Fig. 4:**
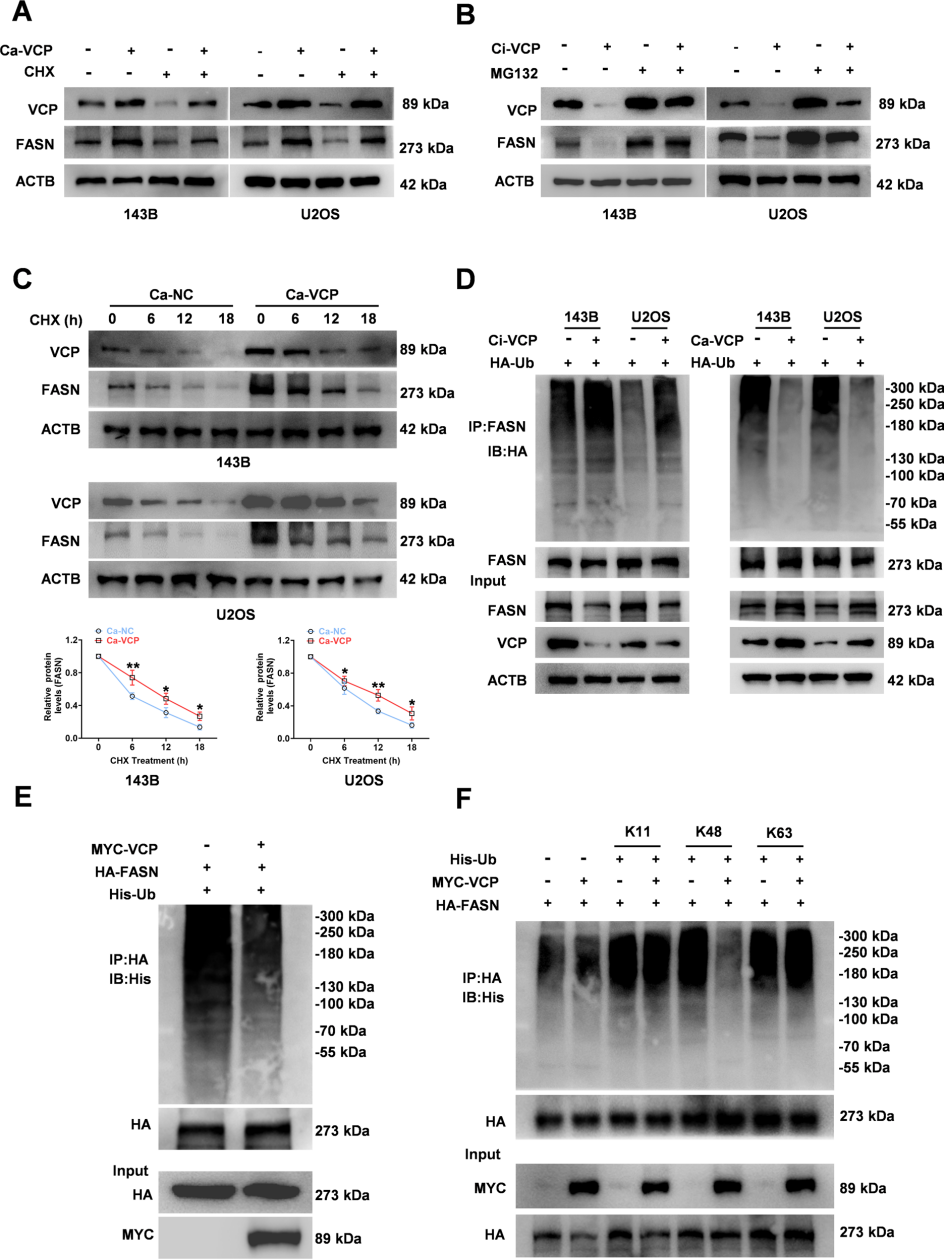
VCP maintains FASN stability in OS. **A**, **B** Western blot assay was utilized to monitor the changes in FASN protein levels, after overexpression (**A**) or knockdown (**B**) of VCP, respectively, treatment with CHX (50 μg/mL, 6 h) or MG132 (20 μM, 6 h) in143B and U2OS cells. **C** Representative images (left) and the quantification (right) of western blot was performed to evaluate the half-life of FASN protein in 143B and U2OS cells. All groups were treated with cycloheximide (CHX, 50 μg/mL), a classic protein synthesis inhibitor, from 0 – 18 h. **D** The total level of FASN ubiquitinated proteins in OS cells was detected by western blot, 143B and U2OS cells were treated with Ca-VCP or Ci-VCP. **E** Levels of exogenous FASN ubiquitination in 293 T cells co-transfected with MYC-tagged VCP, His-tagged Ub and HA-tagged FASN. **F** 293T cells were transiently transfected with plasmids encoding MYC-tagged VCP, HA-tagged FASN, and His-tagged ubiquitin mutants (K11, K48 or K63). FASN ubiquitination was then analyzed by WB.

To further clarify the regulatory mechanism of VCP in FASN protein stabilization, CHX was applied to block mRNA translation in 143B and U2OS cells, and overexpression of VCP still increased the protein expression level of FASN (Fig. 4A). Moreover, inhibition of proteasome activity with MG-132 reversed the VCP silencing-induced downregulation of FASN (Fig. 4B). Furthermore, the half-life of FASN significantly increased in VCP-overexpressing cells (Fig. 4C). Ultimately, we assayed the level of ubiquitination of the FASN protein and found that overexpression of VCP inhibited the ubiquitination of the FASN protein in 143B, U2OS, and 293 T cells, whereas the ubiquitination level of the FASN protein significantly increased in cells with inhibited VCP expression (Fig. 4D, E). Importantly, VCP could remove the K48-linked ubiquitin chains from FASN (Fig. 4F). Taken together, VCP regulates FASN protein levels through the ubiquitin-proteasome degradation pathway.

### VCP inhibits ubiquitination and degradation of FASN proteins by recruiting USP2

**Fig. 5:**
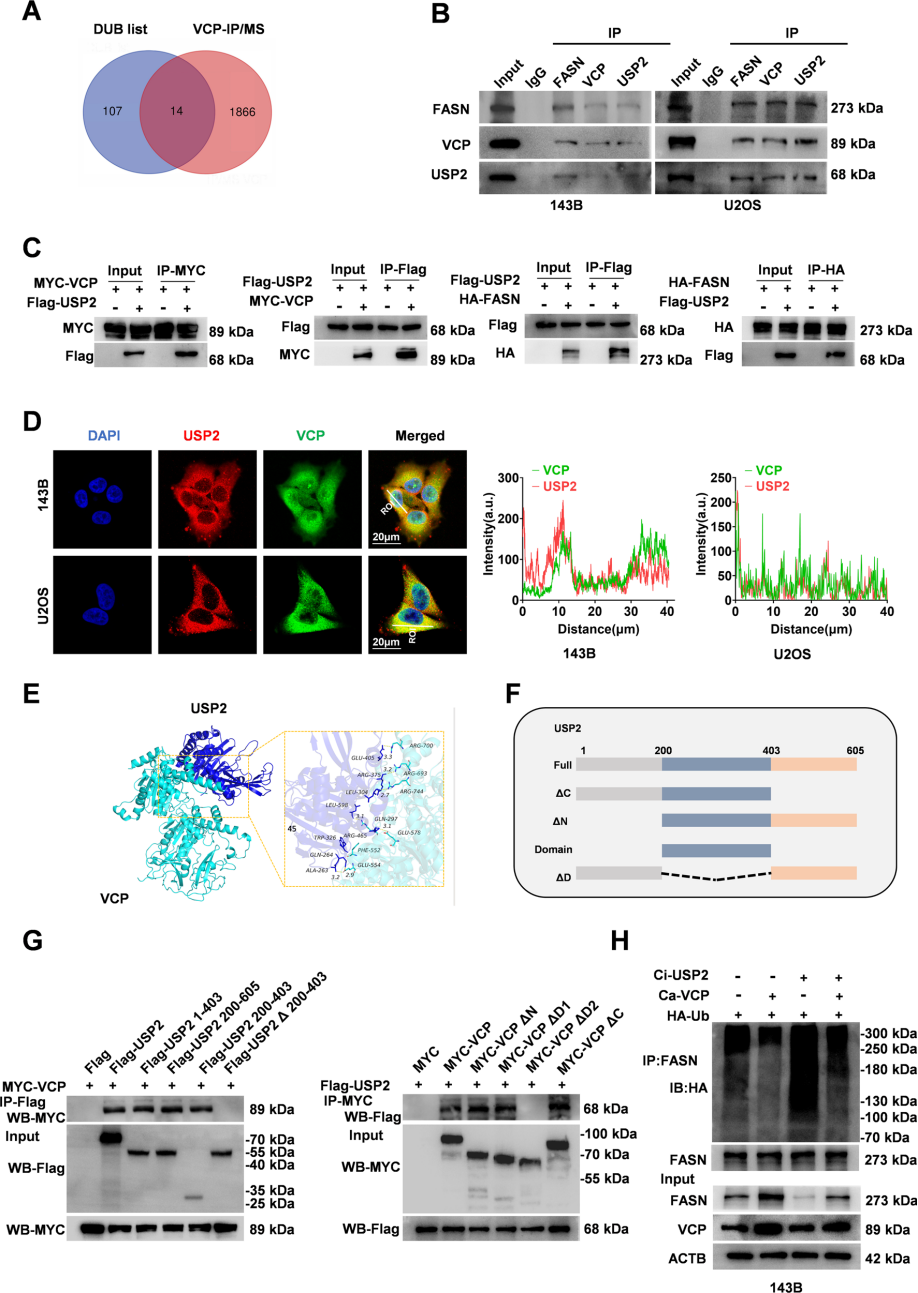
VCP recruits USP2 through deubiquitylation modification to maintain FASN stability. **A** Venn diagram showing 14 candidate proteins obtained, by overlapping VCP-IP/MS, and deubiquitination-associated proteins from web databases. **B** OS cells lysates were immunoprecipitated with FASN, VCP and USP2 antibodies, respectively, to detect endogenous protein interactions. **C** 293 T cell lysates were transfected with HA-tagged FASN, FLAG-tagged USP2 and MYC-tagged VCP plasmids, immunoprecipitated with tagged antibodies, respectively, followed by immunoblotting to detect exogenous protein interactions. **D** Representative images (left) and the quantification (right) of immunofluorescence demonstrate the localization of USP2 and VCP proteins in 143B and U2OS cells. **E** Diagram of VCP and USP2 molecular docking patterns. **F** USP2 truncated structure. The full- length VCP, consisting of 605 amino acids, is divided into four structural domains: 1-403,200-605,200-403 and Δ200-403. **G** 293 T cells were transfected with MYC-labeled full-length VCP and FLAG-labeled full-length USP2 and its truncated structure region. Cell lysates were immunoprecipitated with anti-FLAG antibody or anti-MYC antibody, respectively. Western blot assay was conducted to identify the expression of the MYC probe and FLAG. **H** Total levels of FASN ubiquitinated proteins in OS cells were detected by Western blot, transfected with Ca-VCP, Ci-USP2 or co-transfected.

When bound to deubiquitinating enzymes, VCP can prevent ubiquitination and degradation of target proteins and maintain their stability [33–35]. We detected VCP-interacting proteins by IP-MS (Supplementary Table S3) and obtained a list of deubiquitylation genes from the DUBs database (https://esbl.nhlbi.nih.gov/Databases/KSBP2/Targets/Lists/DUBs/) (Supplementary Table S4). Based on an overlapping analysis of these findings, 14 molecules were identified. Among these, USP2 interacts with FASN and promotes its protein stability by reducing the ubiquitination level of FASN in prostate cancer [36]. Therefore, we speculated that VCP might modify FASN through USP2 deubiquitylation in OS cells. Co-IP analysis revealed that VCP and USP2 were detected in protein precipitates labeled with FASN, while FASN and USP2 were detected in VCP-tagged protein precipitates in OS and 293 T cells (Fig. 5B, C). In addition, confocal microscopy revealed that VCP, USP2, and FASN were localized in the cytoplasm of OS cells. (Fig. 5D). Our study used HDOCK software to predict binding complexes, and the result was a binding score of −265.55 kcal/mol, which can contribute to stable binding interactions (Fig. 5E). Direct physical interaction of VCP with USP2 was observed in 293 T cells, demonstrating that domain 2 of VCP and the amino acid sequence (200-403) of USP2 were critical for their interactions (Fig. 5F, G). To further clarify the interaction between VCP and USP2, we introduced an inhibitor targeting the D2 domain of VCP. CB-5083 (CB) mainly targets the D2 domain of VCP, and has demonstrated significant antiproliferative activity in cancer cells [37, 38]. In addition, the CB-5083 exhibited comparable efficacy in suppressing cellular proliferation in OS cellular model, with a concentration-dependent manner (Supplementary Fig. S6A, B). To confirm whether CB-5083 disrupted the binding of USP2 to the D2 domain of VCP, we conducted CO-IP experiments. The results exhibited that CB-5083 disrupted the VCP-USP2 interaction (Supplementary Fig. S6C). Subsequently, we found that the overexpression of VCP reduced the ubiquitination modification level of FASN and promoted protein stability, whereas the downregulation of USP2 significantly counteracted this effect (Fig. 5H). Accordingly, we speculated that USP2 is a key auxiliary protein for VCP to regulate FASN deubiquitylation and maintain FASN stability.

### VCP facilitates OS autophagy via USP2-mediated deubiquitination of FASN

**Fig. 6:**
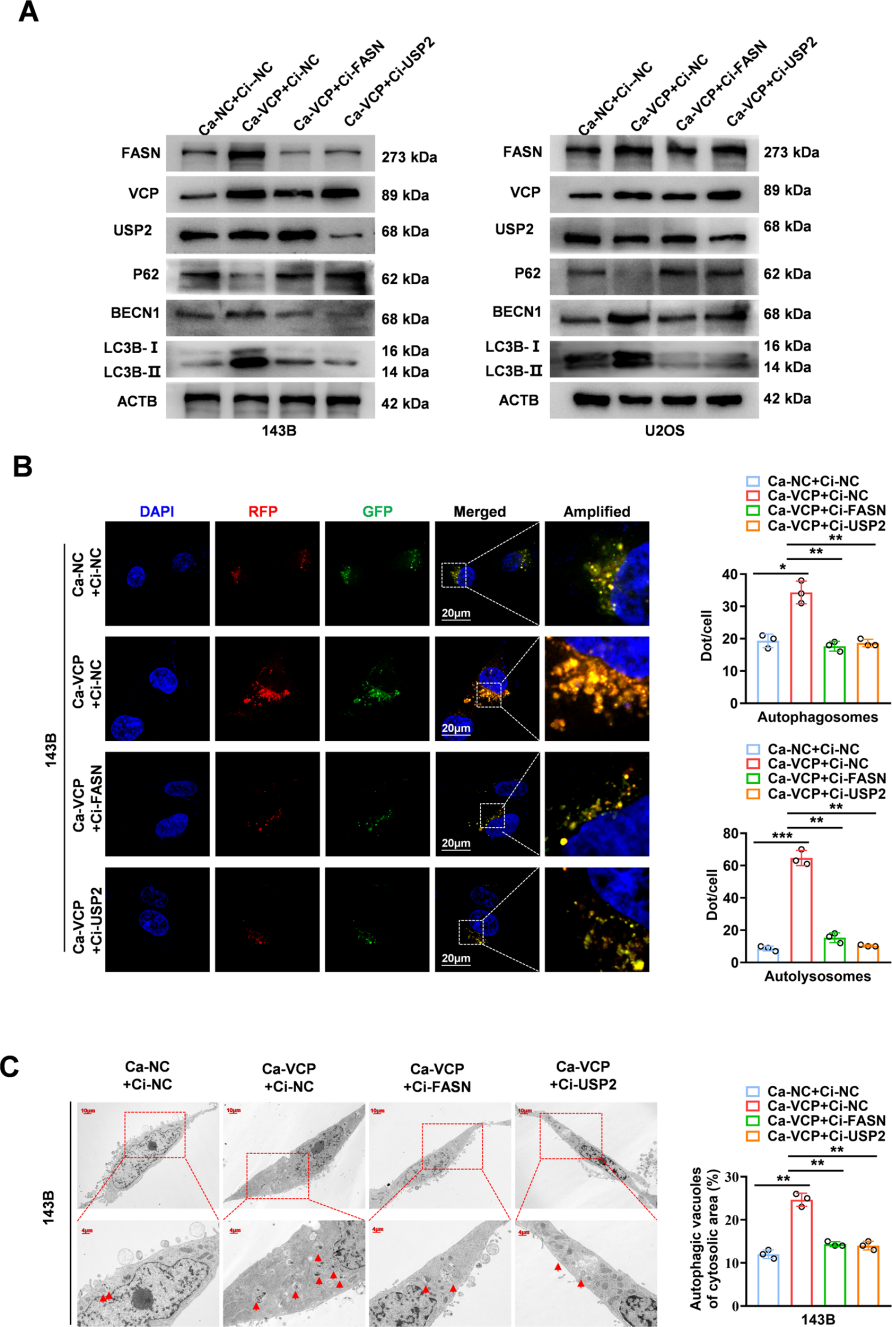
VCP faciliate OS autophagy via USP2-mediated deubiquitination of FASN. **A** Western blot was conducted to analyze the levels of autophagy-related proteins, FASN, and FASN ubiquitination in 143B and U2OS cells stably transfected with Ca-NC, Ca-VCP or co-transfected with Ci-FASN or Ci-USP2. **B** Representative images and quantification revealing the immunofluorescence staining intensity with RFP-GFP-LC3 in 143B cells stably transfected with Ca-NC, Ca-VCP or co-transfected with Ci-FASN or Ci-USP2. Red (RFP + GFP-) puncta represent autolysosomes, and yellow (RFP + GFP + ) puncta represent autophagosomes. Scale bar: 20 µm. **C** Representative images from TEM scanning (left panel) and quantification (right panel) exhibiting autophagic vacuoles in in 143B cells stably transfected with Ca-NC, Ca-VCP or co-transfected with Ci-FASN or Ci-USP2 Scale bar: 20 µm. *P < 0.05 vs. Ca-NC + Ctrl.

To further clarify whether the role of FASN in promoting autophagy in OS cells was affected by the VCP/USP2/FASN axis, we constructed a cell model that overexpressed VCP and downregulated FASN or USP2. Western blot analysis revealed that the levels of LC3B and BECN1 increased in 143B and U2OS cells stably overexpressing VCP. However, the downregulation of FASN or USP2 partly attenuated the autophagic activity induced by VCP overexpression (Fig. 6A). Consistent to these findings, through RFP-GFP-LC3 bifocal fluorescence confocal and TEM experiments, we found that the overexpression of VCP promoted the formation of autophagosomes and autolysosomes and activated autophagy flow in OS cells, whereas the downregulation of FASN or USP2 significantly counteracted the activation effect of VCP on autophagy (Fig. 6B, C).

### VCP harbors oncogenic properties via facilitating USP2-mediated stabilization of FASN

**Fig. 7:**
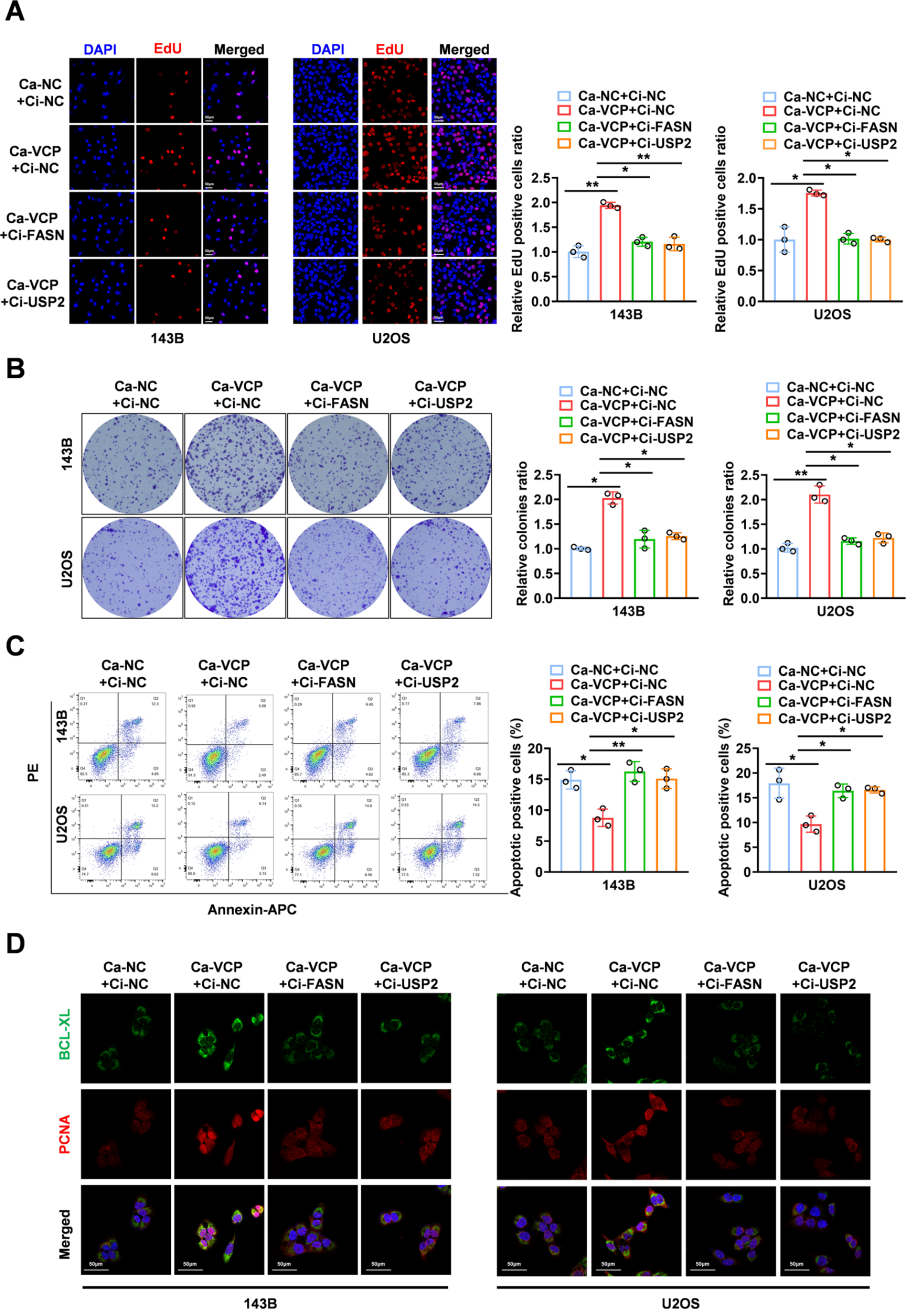
VCP harbors oncogenic properties via facilitating USP2-mediated stabilization of FASN. **A**–**C** Representative images (left) and the quantification (right) of Edu (**A**), colony formation (**B**) and cell apoptosis detection assays showing the growth and the rate of apoptosis of 143B and U2OS cells transfected with Ca-NC, Ca-VCP or co-transfected with Ci-FASN or Ci-USP2. **D** Immunofluorescence assay for the detection of the fluorescence intensity of cell proliferation protein (PCNA) and cellular anti-apoptotic protein (Bcl-xl) in 143B and U2OS cells stably transfected with Ci-ATG5 or Ca-FASN or co-transfected with Ci-ATG5. Scale bar: 50 µm *P < 0.05.

We performed a series of rescue experiments to further evaluate the effect of the VCP/USP2/FASN axis on the biological functions of OS. The results revealed that the stable overexpression of VCP facilitated the viability of OS cells, which was abolished by the transfection of Ci-FASN or Ci-USP2, as demonstrated by Edu and clonal group formation assays (Fig. 7A, B). From flow cytometry and IF data, we observed a significant reduction in the proportion of apoptotic OS cells after VCP overexpression, along with an enhanced fluorescence intensity of the Bcl-xL protein, which could be reduced by inhibiting FASN or USP2 expression (Fig. 7C, D). Thus, we confirmed that VCP regulates FASN stability in conjunction with USP2 to promote OS progression and improve the molecular mechanism of autophagy in OS cells regulated by the VCP/USP2/FASN axis.

### VCP-USP2-FASN axis promotes OS progression in vivo

**Fig. 8:**
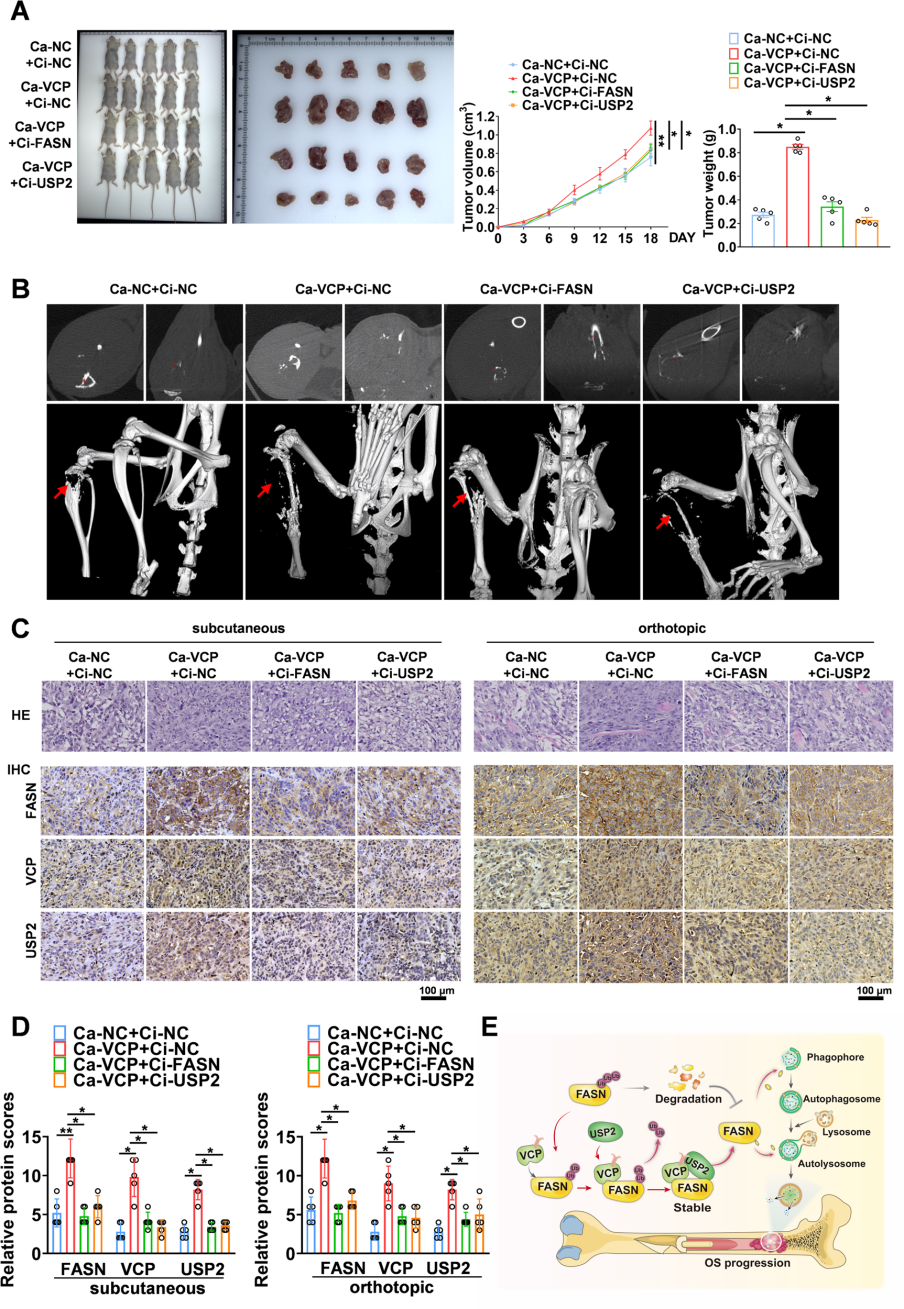
VCP-USP2-FASN axis promotes OS progression in vivo. **A** Representative images of xenograft tumors after established by subcutaneous injection of 143B cells stably transfected with Ca-NC+Ci-NC, Ca-VCP, Ca-VCP+Ci-FASN or Ca-VCP+Ci-USP2 for 2 weeks (left). Quantification of volume and weight of tumor nodules in indicated groups(right). **B** Representative micro-CT images were obtained from in situ tumors after injection of 143B cells stably transfected with Ca-NC+Ci-NC, Ca-VCP, Ca-VCP+Ci-FASN, or Ca-VCP+Ci-USP2 into the proximal tibia for 3 weeks. **C**, **D** HE, IHC and quantitative analyses in subcutaneous (left) and orthotopic (right) xenograft models revealed the tumor cells and the differences in FASN, VCP, and USP2 protein expression. Scale bar: 50 μm. **E** Mechanisms underlying FASN-promoted autophagy and OS progression. *P < 0.05.

We established an OS mouse model using 143B cells to evaluate the role of the VCP/USP2/FASN axis in promoting OS progression in vivo. The results of the animal model suggest that overexpression of VCP significantly promoted tumor size and weight in the subcutaneous xenograft and orthotopic transplantation models, and, crucially, the promoting effect of VCP was significantly limited after the downregulation of FASN or USP2, respectively (Fig. 8A). Moreover, the results of micro-CT analysis showed that more bone erosion was observed in the groups injected with Ca-VCP cells compared to that in the control cells, and the downregulation of FASN or USP2 clearly prevented the beneficial effects of VCP elevation (Fig. 8B). Hematoxylin and eosin (H&E) staining identified an increase in tumor cells in the VCP-overexpressing group, while the immunohistochemical (IHC) analysis showed that in the VCP gene activation group, the expression of FASN, VCP, and USP2 in tumor cells was relatively increased (Fig. 8C, D). These results provide convincing evidence of the oncogenic role of the VCP/USP2/FASN axis in OS.

Targeting FASN or USP2 could potentially disrupt essential fatty acid metabolism or ubiquitin homeostasis in healthy cells. Whether silencing FASN in osteoblasts affects cell viability remains to be investigated. First, we detected the protein expression of FASN and USP2 in OS cell lines and hFOB1.19 cells (normal osteoblasts). The western blot results revealed that FASN and USP2 were highly expressed in OS cell lines compared to hFOB 1.19 cells (Supplementary Fig. S7A). Furthermore, we knocked down the expression of FASN or USP2 in hFOB 1.19 cells via CRISPRi method (Supplementary Fig. S7B). The CCK-8 assays showed that silencing FASN had minimal impact on proliferation of osteoblast (Supplementary Fig. S7C). This minimal impact may be attributed to the fact that de novo fatty acid synthesis occurs at low levels because dietary supply usually meets cellular demands in healthy cells [39]. Thus, disrupting FASN in healthy cells like osteoblasts does not significantly affect cell viability. However, USP2 knockdown significantly impaired hFOB 1.19 cells viability (Supplementary Fig. S7D).

In conclusion, FASN plays an oncogenic role in OS progression by activating autophagy. Mechanistically, VCP interacted with and inhibited FASN degradation, leading to autophagy and tumorigenesis in OS cell (Fig. 8E). As a deubiquitinating enzyme, USP2 is recruited by VCP to inhibit the ubiquitination and degradation of FASN, thereby promoting FASN-mediated autophagy and OS progression. FASN may serve as a possible diagnostic and prognostic biomarker, as well as a potential target for future OS therapies.

## Discussion

Impairments in the physiological activation, assembly, and functional pathways of autophagy have been increasingly observed in various cancers [40]. Fatty acids activate autophagy by providing sufficient energy and raw materials for phagocytic vesicle expansion and autophagic lysosome formation [41]. Furthermore, FASN may serve as an oncogenic factor owing to its role in regulating fatty acid synthesis and driving aberrant lipogenesis in cancer cells [42]. However, the origin and regulatory mechanisms of fatty acids in autophagy activation, particularly in bone tumors, have not been completely clarified. Our study revealed the important role of FASN in autophagy. Ectopic expression of FASN promoted the formation of autophagosomes and autolysosomes, whereas FASN downregulation inhibited early and late autophagy, resulting in a significant reduction in autophagosomes and autolysosomes. Further research revealed the significance of FASN-activated autophagy in OS progression and lung metastasis. The malignant behavior observed in OS mediated by FASN was drastically suppressed by inhibiting autophagy activation through downregulation of ATG5. Furthermore, ATG5 stabilization regulates glycolytic reprogramming and progression of OS [25]. According to these studies, autophagy and FASN are predictive biomarkers for patients with OS. From a clinical perspective, it is important to develop more effective and highly specific drugs targeting autophagy or FASN.

Interestingly, our results showed that overexpression or downregulation of VCP significantly increased or decreased FASN protein levels, respectively, but had no effect on mRNA levels. These findings motivated us to focus on the post-translational modification of the FASN protein. VCP is a member of the AAA ATPase protein family that plays crucial roles in diverse cellular processes, such as protein degradation, intracellular membrane fusion, DNA repair and replication, cell cycle regulation, and autophagy activation [43]. Structurally, each VCP subunit contains a regulatory N-terminal domain and two ATPase domains (D1 and D2), which enable VCP to interact with >30 different cellular proteins and participate in a variety of cellular functions [44]. Furthermore, VCP is involved in multiple disease states, including neurodegenerative diseases, and several tumor types, such as squamous cell carcinoma, prostate cancer, esophageal cancer, and colorectal tumors [35]. Our results demonstrated that stress stimulation increases the binding of the D2 structural domain of VCP to FASN proteins, ultimately promoting autophagy in OS cells. These findings provide better insights into the physiological functions of VCP substrates. However, further in vitro and in vivo studies are required to fully understand the regulation of autophagy triggered by interactions between VCP and FASN.

VCP can inhibit the ubiquitination and degradation of target proteins and maintain their stability through a number of specialized adaptor proteins [43]. To investigate the mechanisms by which VCP regulates FASN, we focused on the USP2 protein. USP2, a well-characterized member of the USP family, is a cysteine protease and a key regulator of physiological and pathological processes, such as protein metabolism, cell cycle regulation, DNA damage repair, and gene transcription [45]. Moreover, USP2 interacts with and stabilizes MDM2, PD-L1, Smad4, and β-linked protein [46]. In our study. IP/MS and ubiquitination assays confirmed that VCP promotes FASN deubiquitination by recruiting USP2 to maintain FASN stability. USP2 downregulation disrupts the role of VCP in maintaining FASN stability, suggesting that USP2 plays a central role in promoting FASN deubiquitination. In addition, USP2 and FASN expression levels were elevated with higher tumor malignancy in gliomas, according to the WHO classification [47]. Although multidisciplinary research has significantly improved our understanding of the ubiquitin mechanisms over the last few years, further studies on the mechanism and transformation of ubiquitin are essential.

The VCP/USP2/FASN axis could emerge as a compelling therapeutic target in cancer treatment. Several FASN inhibitors, such as cerulenin, GSK2194069, and orlistat, which target the KS, KR, and TE domains respectively, have been identified [48, 49]. FT-4101, another FASN inhibitor, is currently under clinical evaluation [50]. These promising findings from preclinical and clinical studies have driven the search for potential FASN inhibitors. To facilitate clinical translation, a structure-guided approach recently led to the discovery of FASstatin, a small-molecule inhibitor of FASN. It has great promise in improving nonalcoholic fatty liver disease and nonalcoholic steatohepatitis, with favorable safety, tolerability, and pharmacokinetics [51]. Another significant advancement is TVB-2640, the first orally bioavailable FASN inhibitor to enter Phase II clinical trials [52]. In addition, TVB3664, when combined with cabozantinib, has potential in downregulating multiple cancer-associated pathways, including AKT/mTOR, and in inhibiting cell proliferation [53].

The exploration of deubiquitinating enzymes (DUBs) as drug targets is still nascent. Although numerous highly selective inhibitors have been developed, few have entered clinical trials [54, 55]. Notably, ML364, a dual-target inhibitor of USP2 and USP8, has garnered attention for its ability to activate p53 by destabilizing VPRBP, thus inhibiting tumor growth [46, 56, 57]. LLK203 is a more potent dual-target inhibitor that downregulates several breast cancers–dependent proteins and inhibits cell proliferation; However, the low bioavailability of it limits its broader application [58]. Despite the large number of proteasome inhibitors utilized in the clinical setting, resistance to treatment is common, and their efficacy against solid tumors is limited [35]. In contrast, VCP inhibitors exhibit significant effects against both hematologic and solid malignancies. Among ATP-competitive inhibitors, N2, N4-dibenzylquinazoline-2,4-diamine (DBeQ) induces selective apoptosis in breast, colon, and bladder cancer cells [59, 60]. CB-5083, a VCP inhibitor with high specificity for the D2 domain, has demonstrated good solubility, oral bioavailability, and high uptake in tumor tissues [61, 62]. Subsequently, more specific ATP-competitive inhibitors, ML-240 and ML-241, were developed to target the D2 ATPase structural domain of VCP; however, these compounds exhibit poor efficacy in vivo and lack favorable pharmacological properties [63].Given the challenges of off-target toxicity and drug resistance, exploring combination therapies and targeted treatments have become essential. Nanomedicines, which promote T-cell infiltration, can elicit a robust and sustained anti-tumor immune response [37]. These versatile nanomedicines can be tailored to remodel the tumor microenvironment (TME) and optimize combination therapies to enhance efficacy and safety [64]. Ultimately, advancing current inhibitors, integrating combination strategies, and discovering new drug targets are critical priorities in cancer therapy.

In conclusion, our data demonstrated that FASN plays an oncogenic role in OS progression by activating autophagy. Mechanistically, VCP interacted with and inhibited FASN degradation, leading to autophagy and tumorigenesis in OS cells. As a deubiquitinating enzyme, USP2 is recruited by VCP to inhibit the ubiquitination degradation of FASN, thereby promoting FASN-mediated autophagy and OS progression (Fig. 8E). Our findings suggest that FASN may serve as a possible diagnostic and prognostic biomarker, as well as a potential target for new therapies for OS in the future.

## Supplementary information

Supplementary Figures

original western blots

Supplementary Tables

## Data Availability

The data supporting the conclusions of this article are included in this article and its additional files.
