# Supplementary material for: VCP enhances autophagy-related osteosarcoma progression by recruiting USP2 to inhibit ubiquitination and degradation of FASN
Source: Cell Death Dis. 2024 Nov 3;15(11):788. doi: 10.1038/s41419-024-07168-6 (PMC11532476; doi:10.1038/s41419-024-07168-6)
Supplement: Supplementary file 1 — Supplementary Figures [file 41419_2024_7168_MOESM1_ESM.pdf]

Supplementary Materials

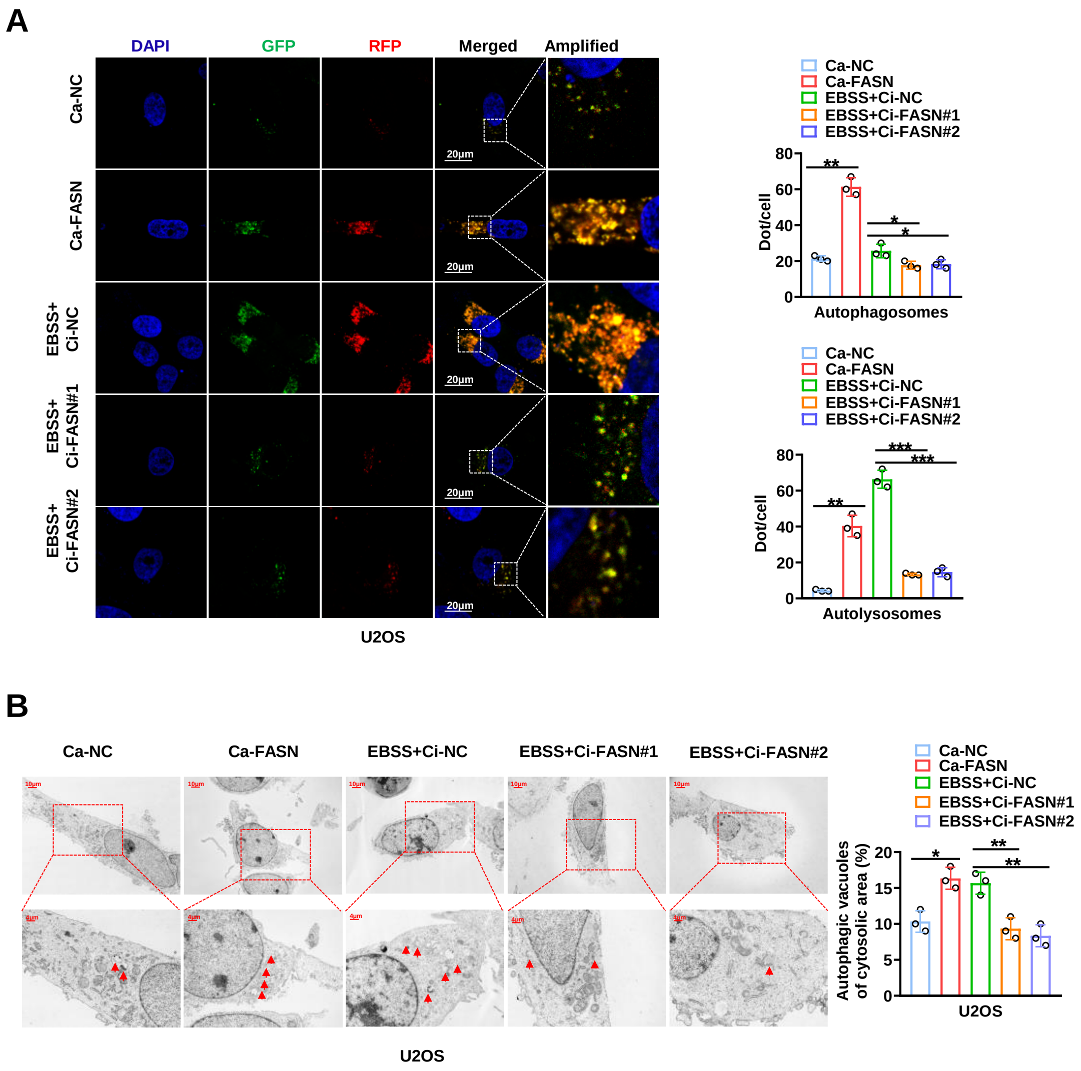

Fig. S1 FASN facilitates autophagy in OS cells

(A) Representative images (left) and quantified results (right) showing the intensity of RFP-GFP-LC3 immunofluorescence staining in U2OS cells stably transfected with Ca-FASN and treated with Ci-FASN#1 or Ci-FASN#2 and induced with EBSS. Red and yellow dots represent autolysosomes and autophagosomes, respectively. Scale bar: 20 μm. **(B)** TEM images of autophagic vacuoles in U2OS cells treated with Ca-FASN, Ci-FASN#1, or Ci-

FASN#2 and induced with EBSS. *P < 0.05.

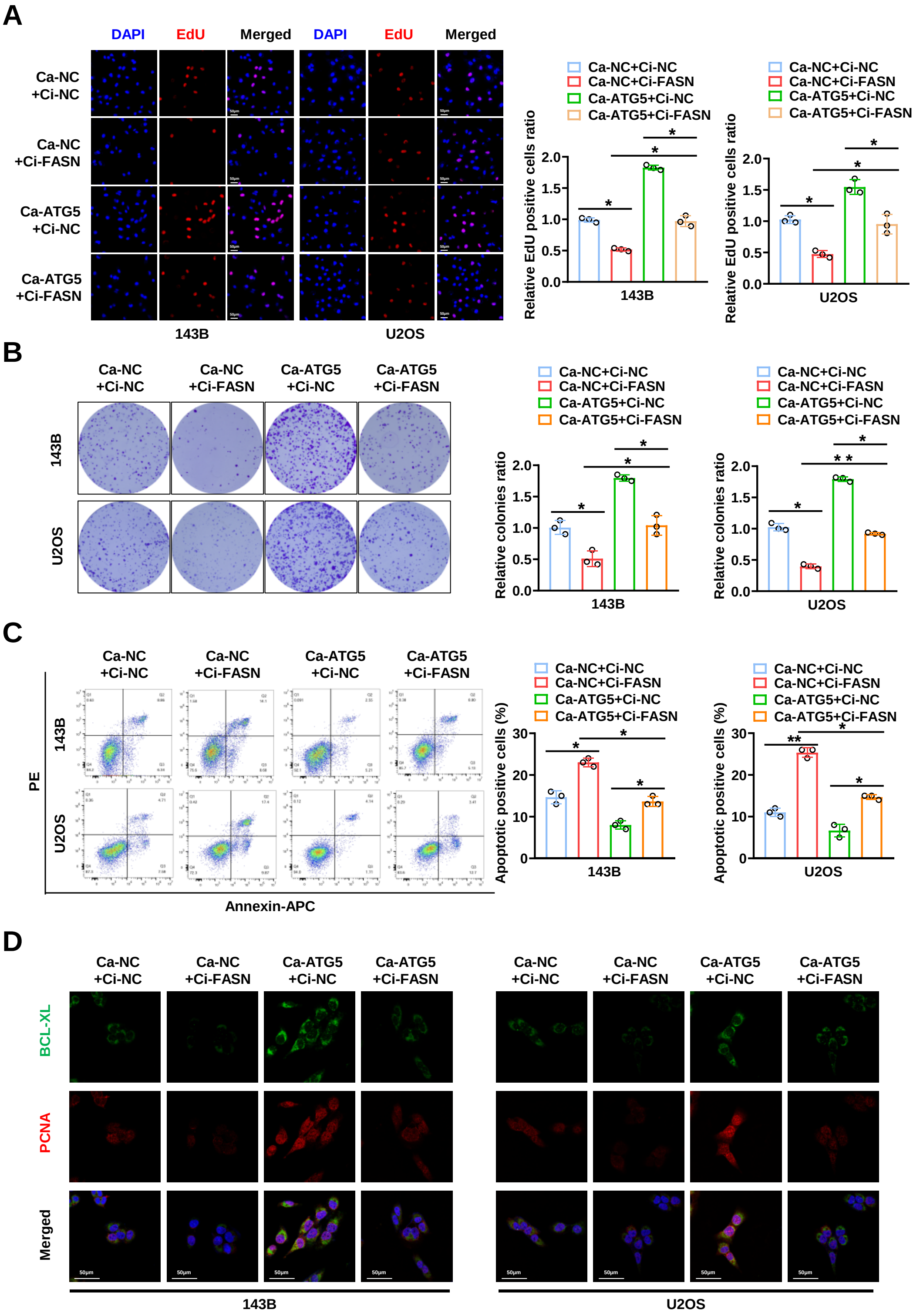

Fig. S2 FASN promotes tumorigenesis in an autophagy-dependent manner

(A-C) Representative images (left) and quantified results (right) of Edu, colony formations, and flow cytometry assays showing the growth and apoptotic rate of 143B and U2OS cells stably transfected with Ca-ATG5 or Ci-FASN or co-transfected with Ca-ATG5. **(D)** Immunofluorescence assay for the detection of the fluorescence intensity of cell proliferation protein (PCNA) and cellular anti-apoptotic protein (Bcl-xl) in 143B and U2OS cells stably transfected with Ca-ATG5 or Ci-FASN or co-transfected with Ca-ATG5. Scale bar: 50 μm *$P < 0.05$.

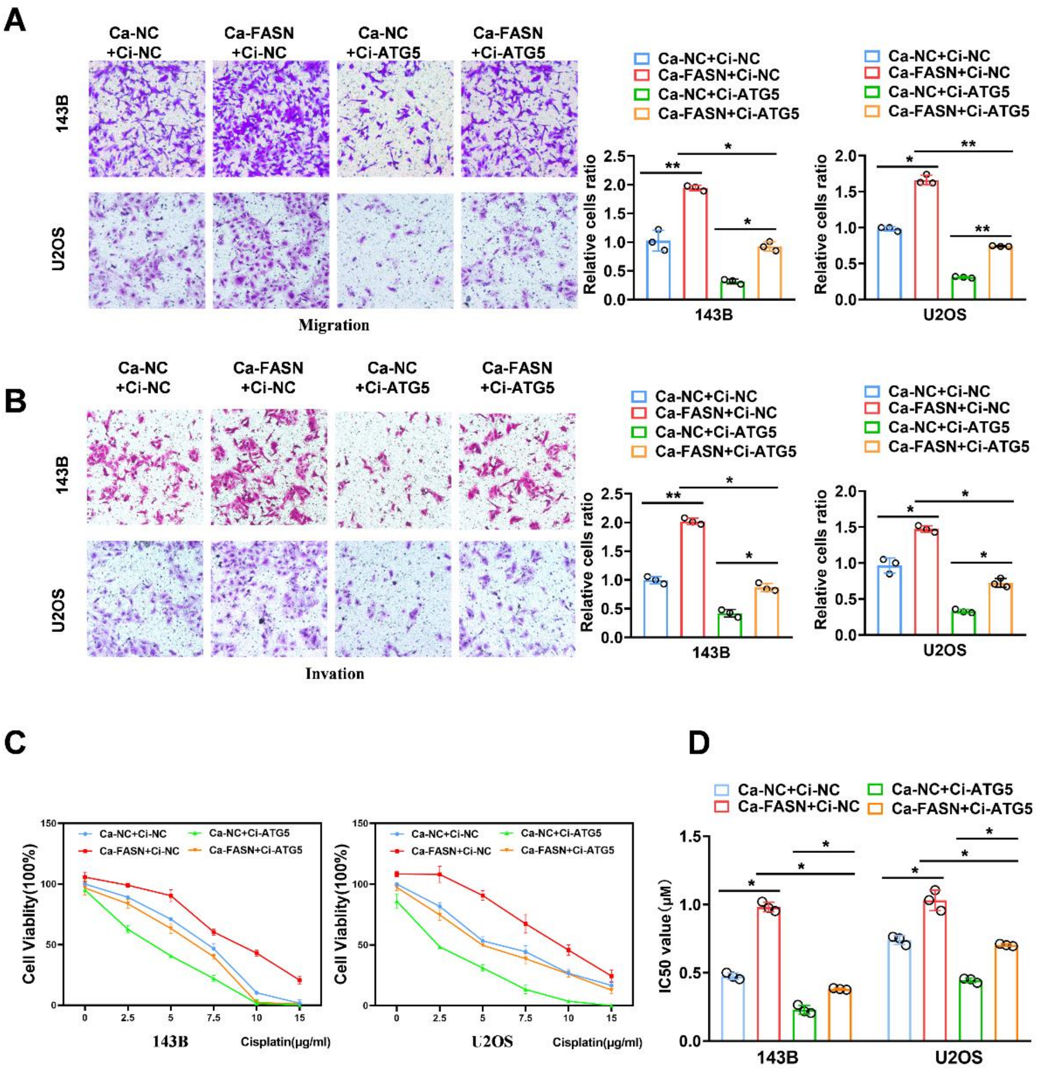

Fig. S3 FASN promotes invasion, migration, and resistance to chemotherapy of OS cells in an autophagy-dependent manner

(A-D) Representative images (left) and the quantification (right) of Transwell and CCK-8 experiments showing the invasion, migration and resistance to chemotherapy capacity of 143B and U2OS cells stably transfected with Ci-ATG5 or Ca-FASN or co-transfected with Ci-ATG5. *P < 0.05.

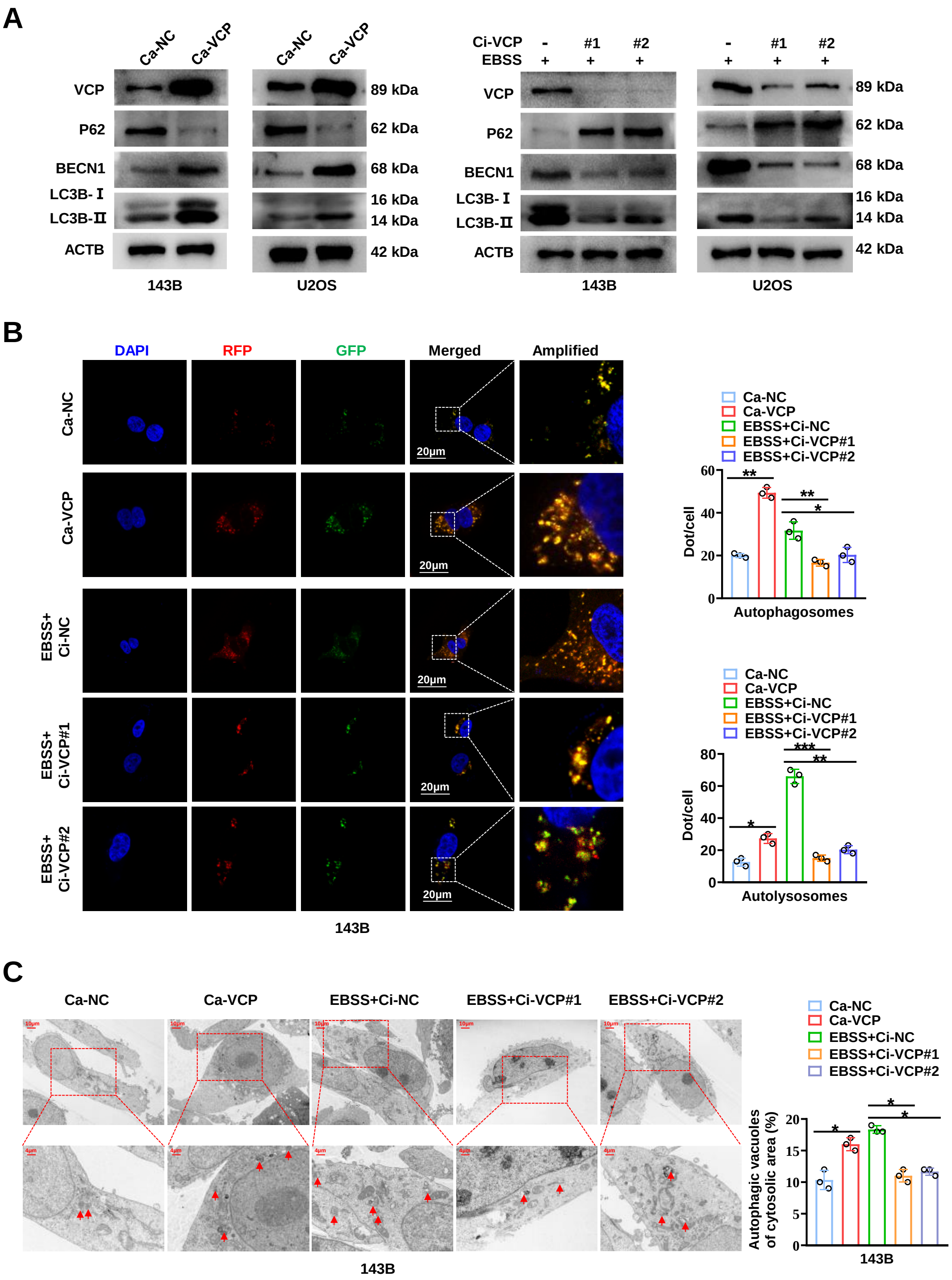

Fig. S4 VCP facilitates autophagy in OS

(A) Western blot images showing the expression of autophagy-associated

proteins in 143B and U2OS cells treated with Ca-VCP, Ci-VCP#1, or Ci-VCP#2 and induced with EBSS. **(B)** Representative images (left) and quantitative results (right) showing the intensity of RFP-GFP-LC3 immunofluorescence staining in 143B cells stably transfected with Ca-VCP and treated with Ci-VCP#1 or Ci-VCP#2 and induced with EBSS. Red and yellow dots represent autolysosomes and autophagosomes, respectively. Scale bar: 20 µm. **(C)** TEM images of autophagic vacuoles in 143B cells treated with Ca-VCP and Ci-VCP#1 or Ci-VCP#2 and induced with EBSS. *P < 0.05.

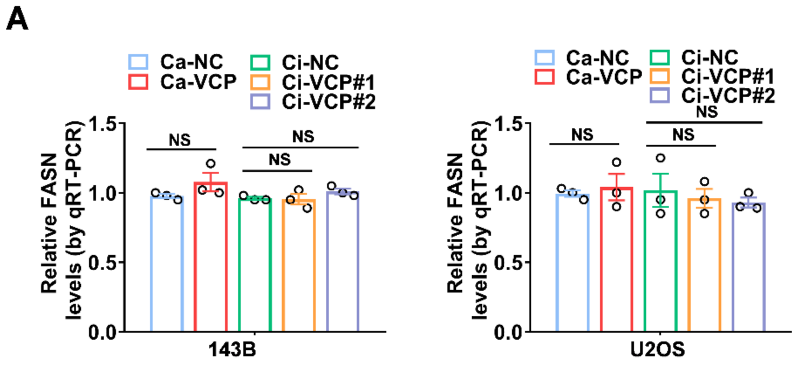

Fig. S5 Overexpressing or knocking down VCP does not affect FASN mRNA levels

(A) qRT-PCR analysis results showing changes in FASN mRNA levels upon VCP overexpression or knockdown.

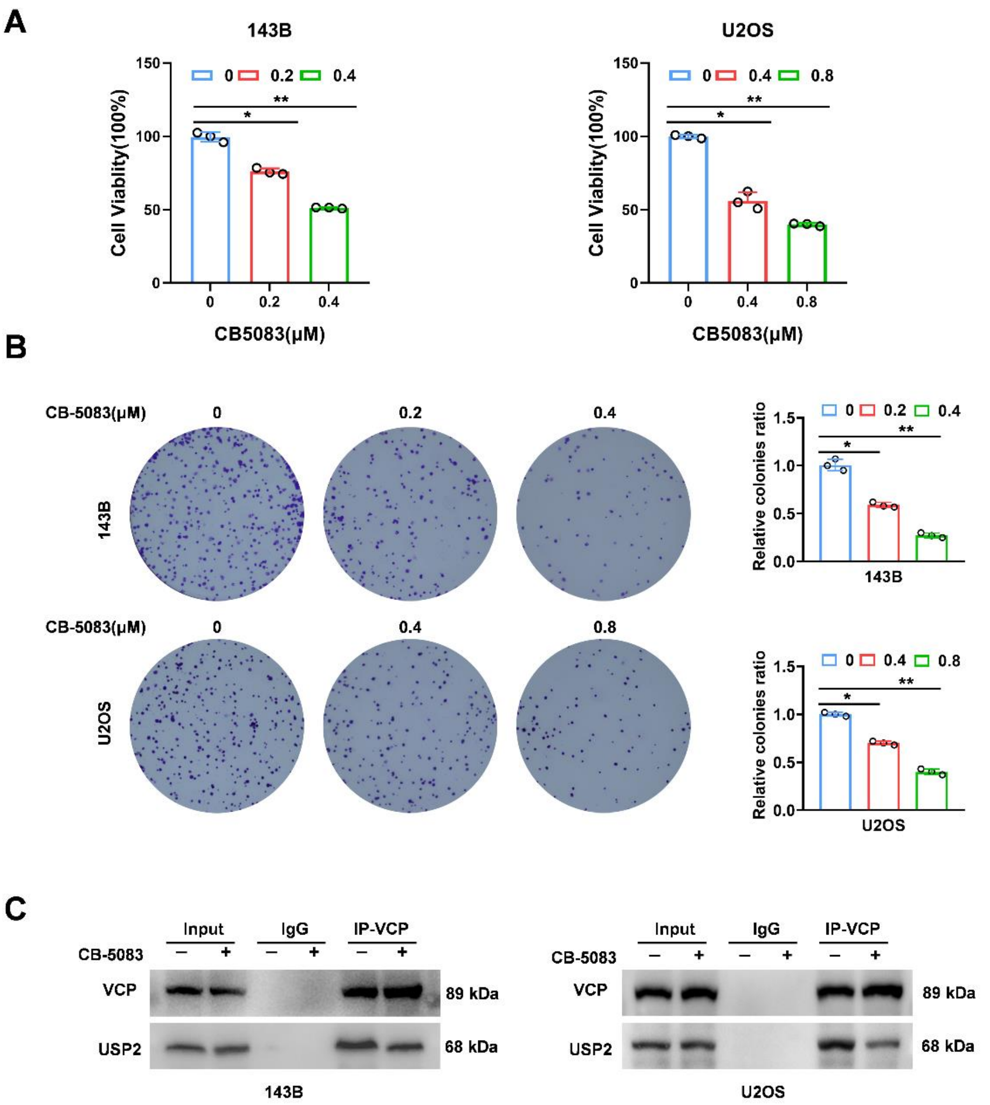

Fig. S6 CB-5083 inhibits OS cells growth and disrupts the interaction between VCP and USP2

(A-B) CCK-8 and colony formations assays evaluated the growth rate of 143B and U2OS cells treatment with CB-5083. **(C)** CO-IP assay was utilized to measure the potency of VCP interactions with USP2, following treatment with CB-5083 for 24 hours. *P < 0.05.

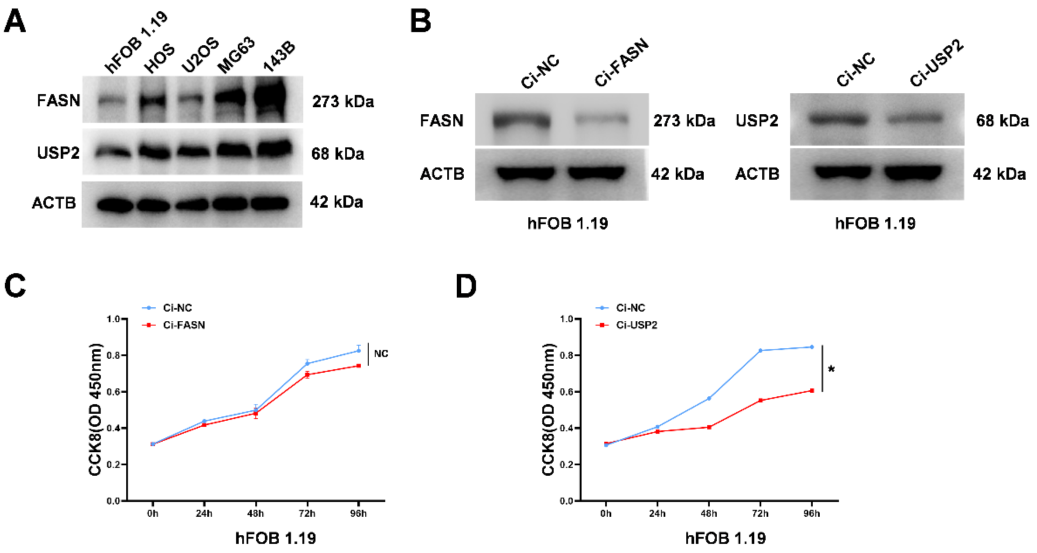

Fig. S7 The effect of FASN or USP2 knockdown on the viability of normal cells

(A) Western blot analysis was performed to assess the expression levels of FASN and USP2 in osteosarcoma cell lines, compared to normal cells. **(B)** Western blot analysis confirmed the knockdown efficiency of FASN or USP2 in normal cells. **(C-D)** The CCK-8 assay evaluated the changes in cell viability of normal cells following the knockdown of FASN or USP2.
