# Supplementary figures and images for: VCP enhances autophagy-related osteosarcoma progression by recruiting USP2 to inhibit ubiquitination and degradation of FASN
Source: Cell Death Dis. 2024 Nov 3;15(11):788. doi: 10.1038/s41419-024-07168-6 (PMC11532476; doi:10.1038/s41419-024-07168-6)

Fig. 1

A

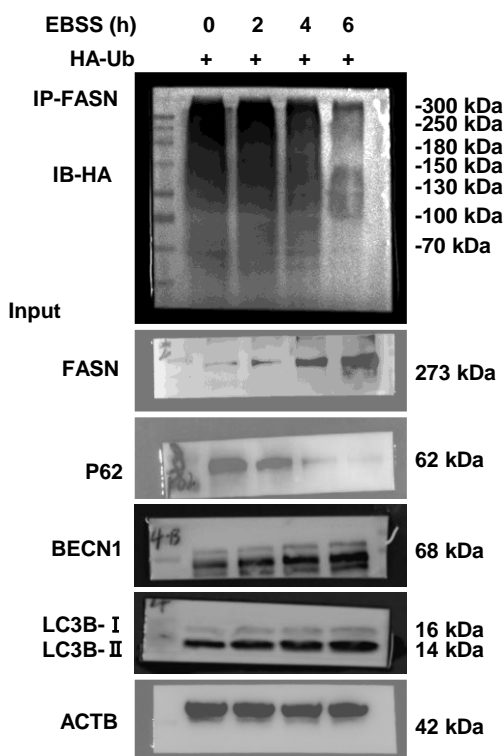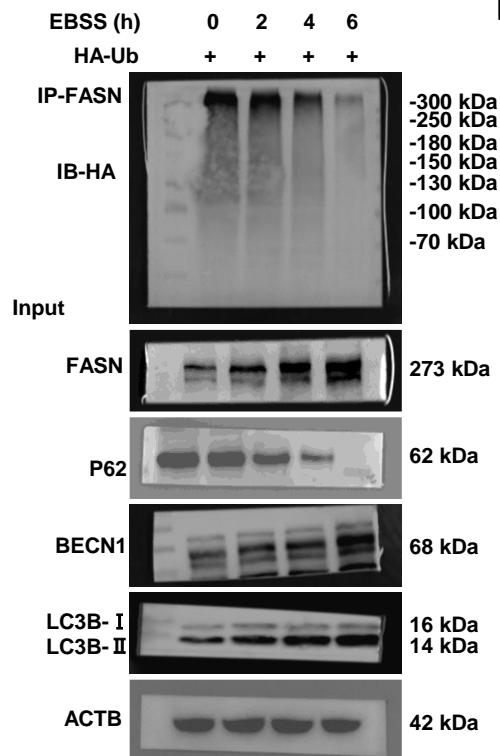

D

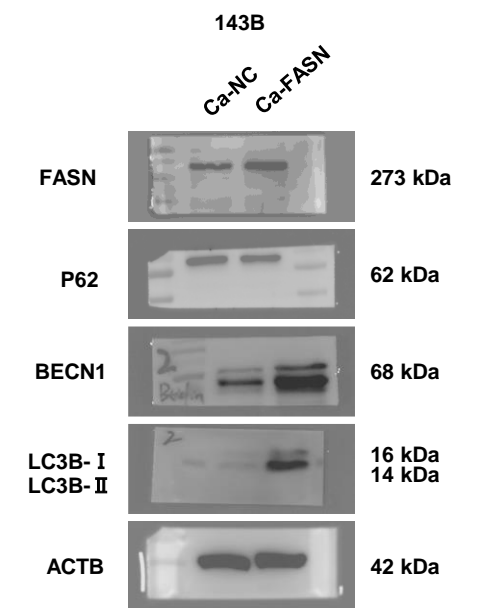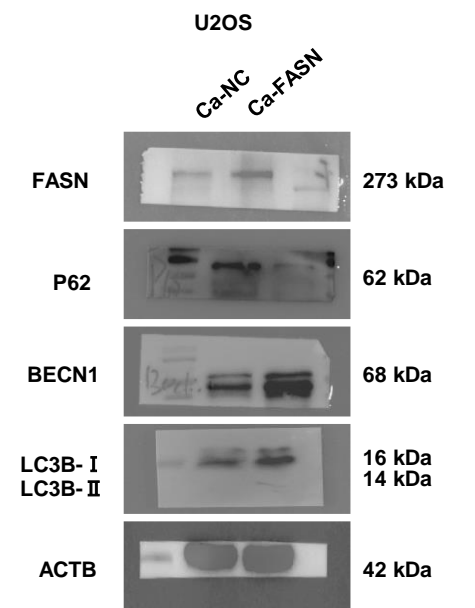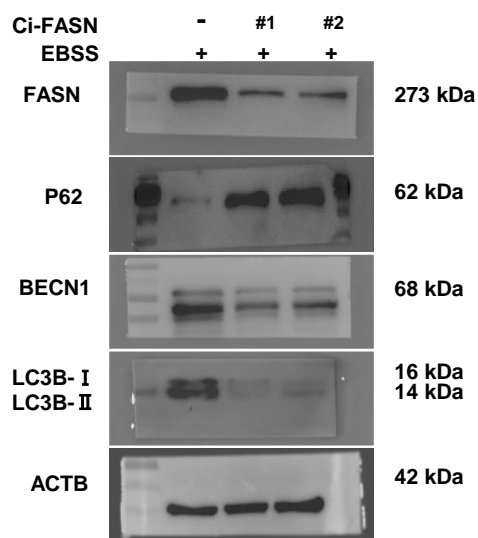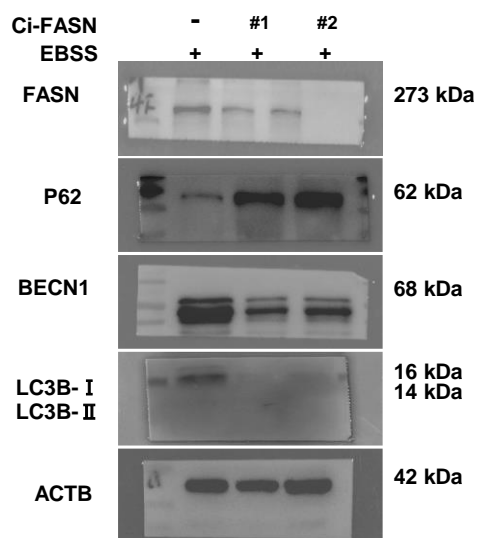

C

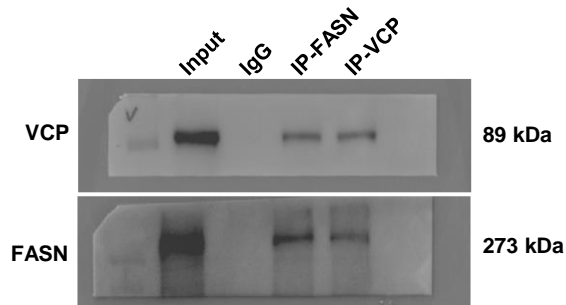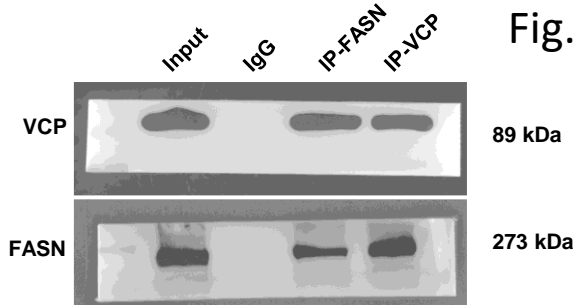

D

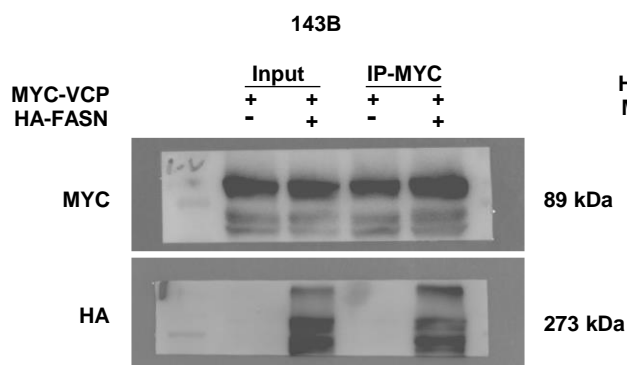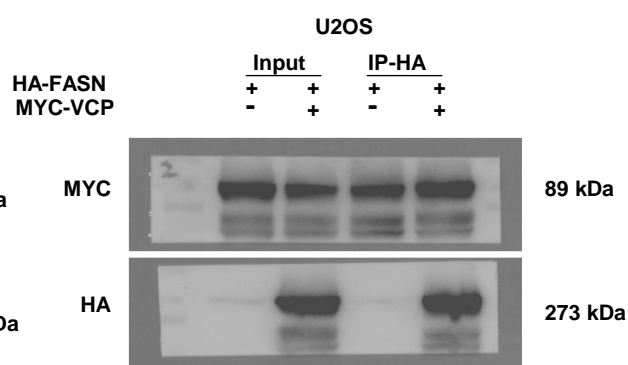

H

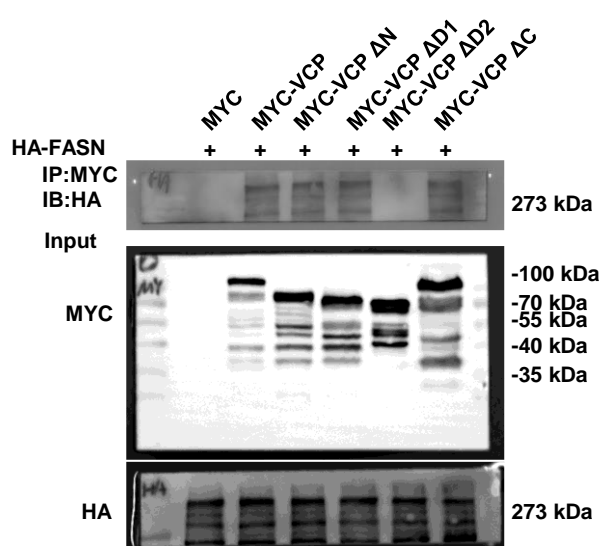

I

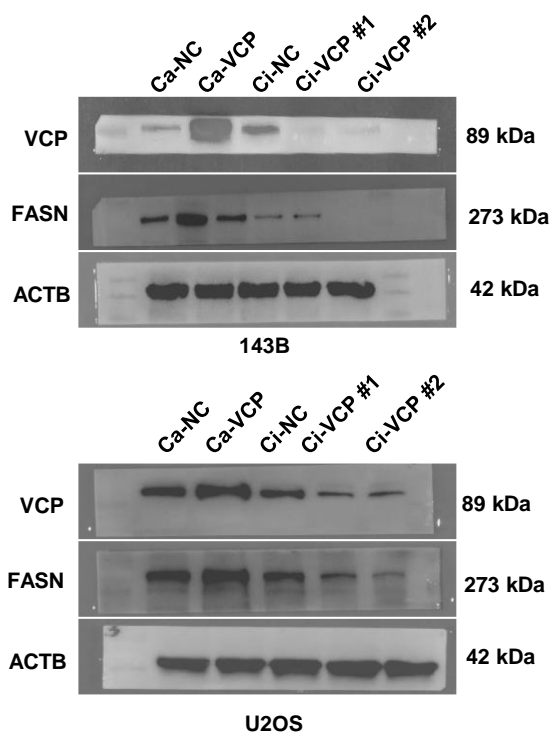

J

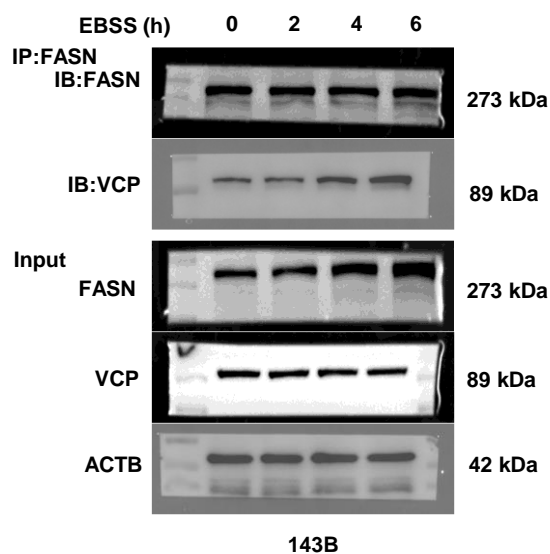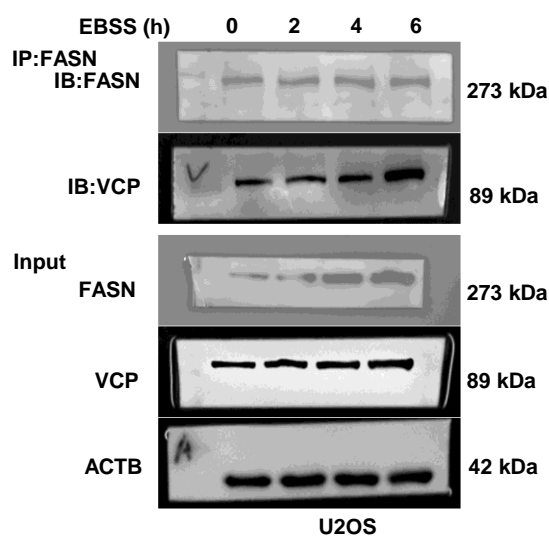

Fig. 4

**A**

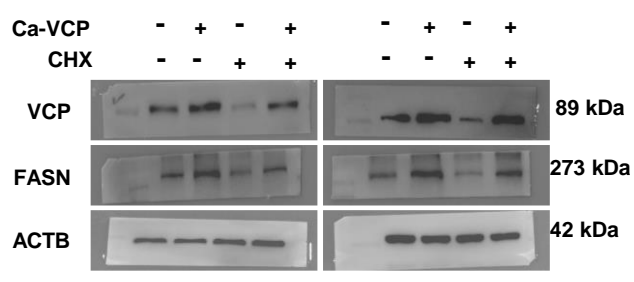

**B**

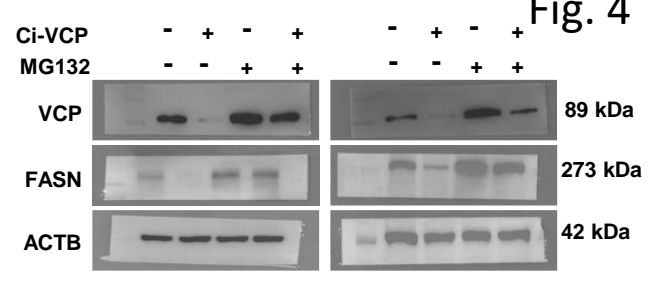

**C**

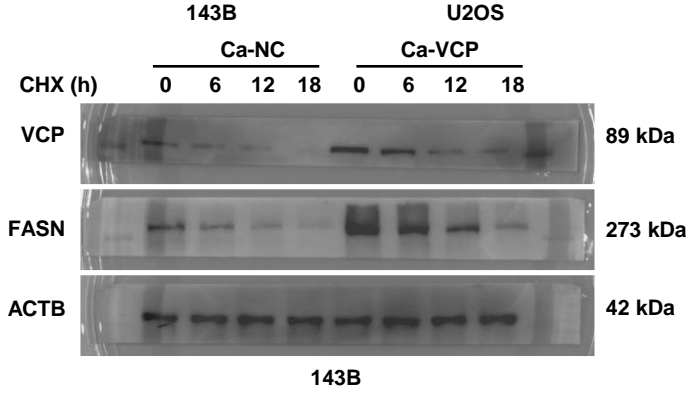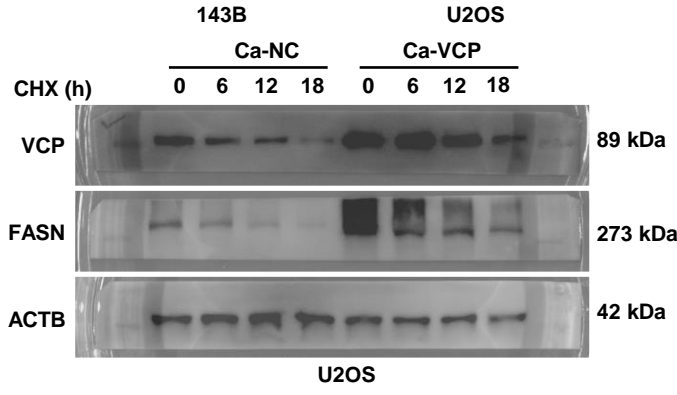

**D**

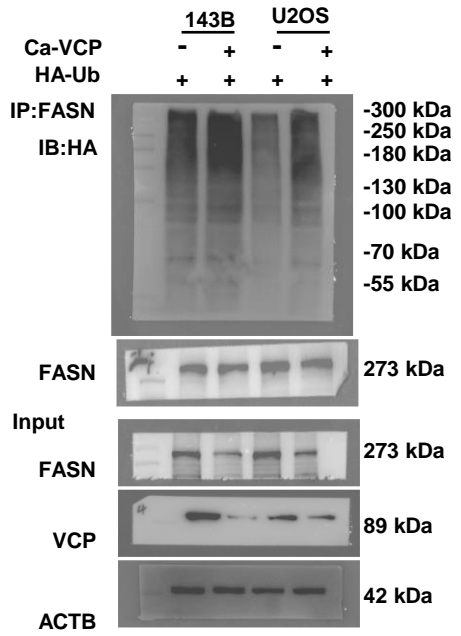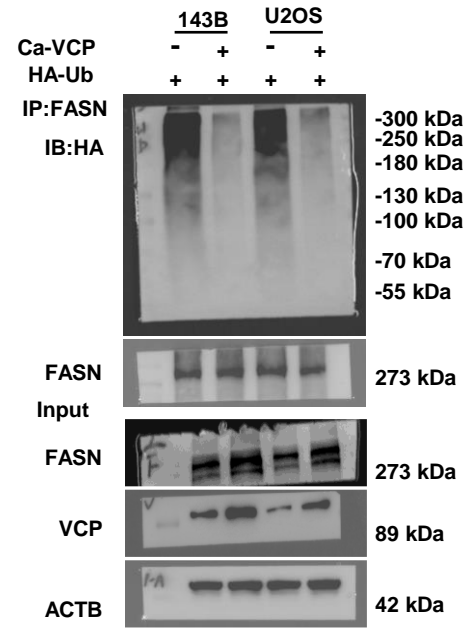

**E**

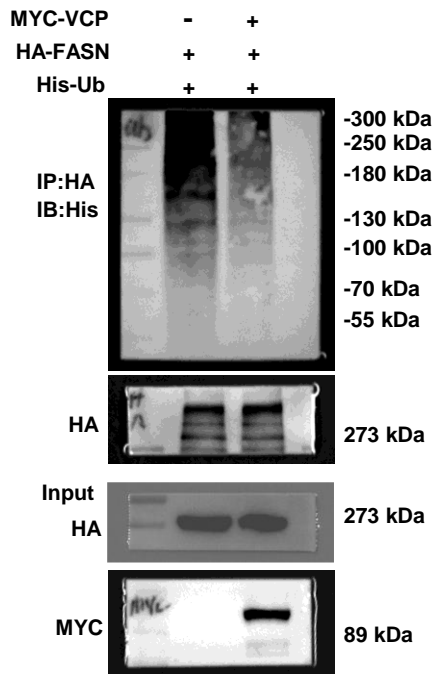

**F**

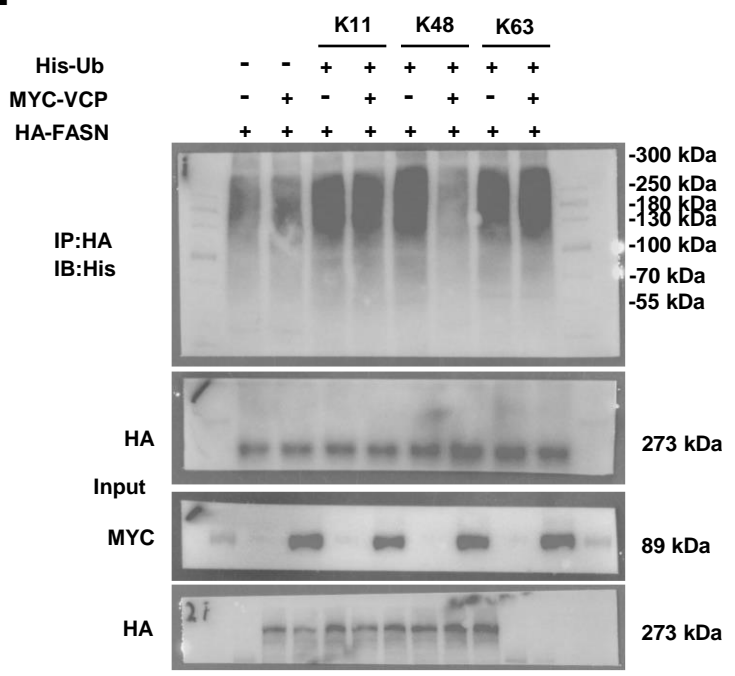

**B**

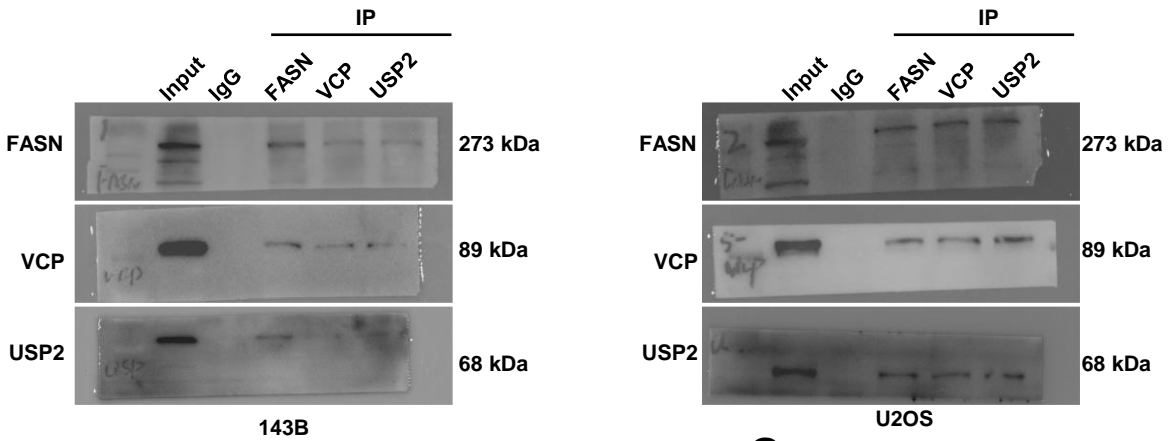

**C**

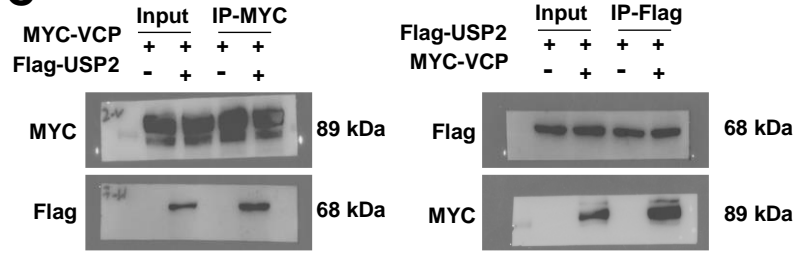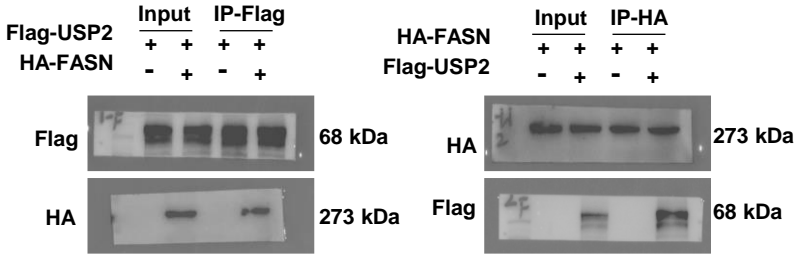

**H**

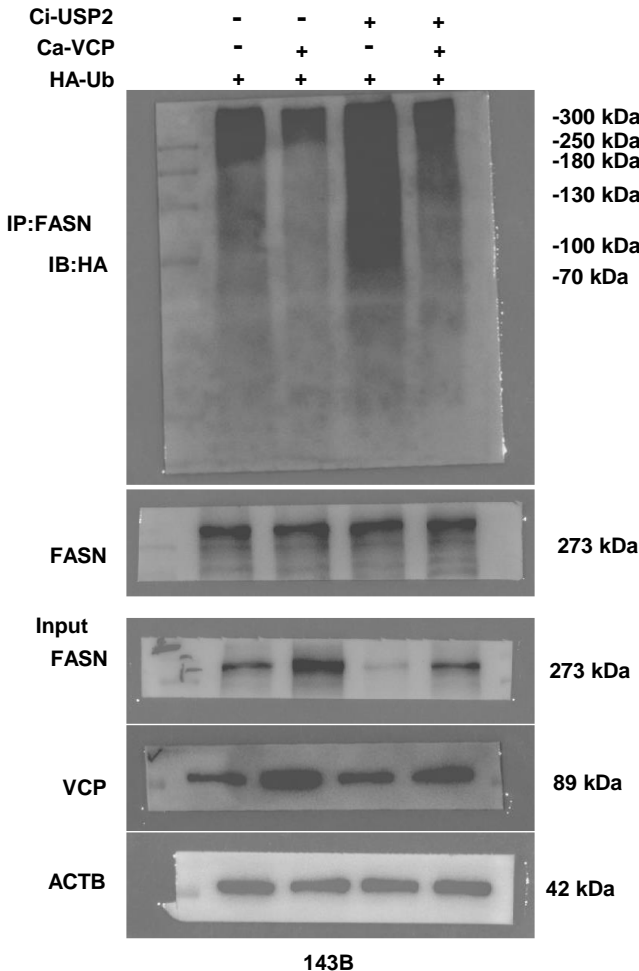

**G**

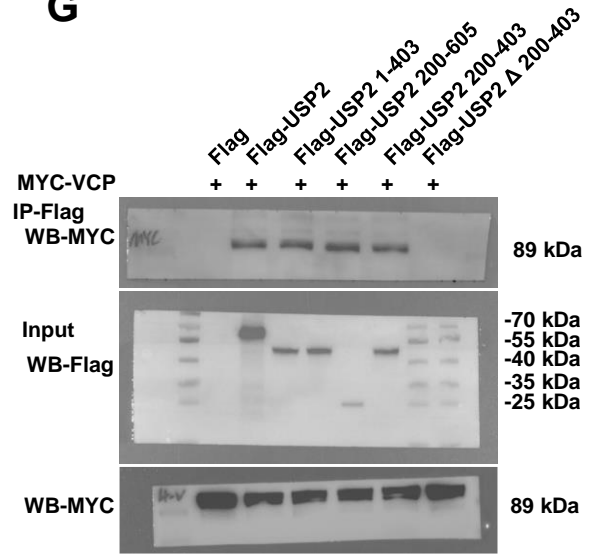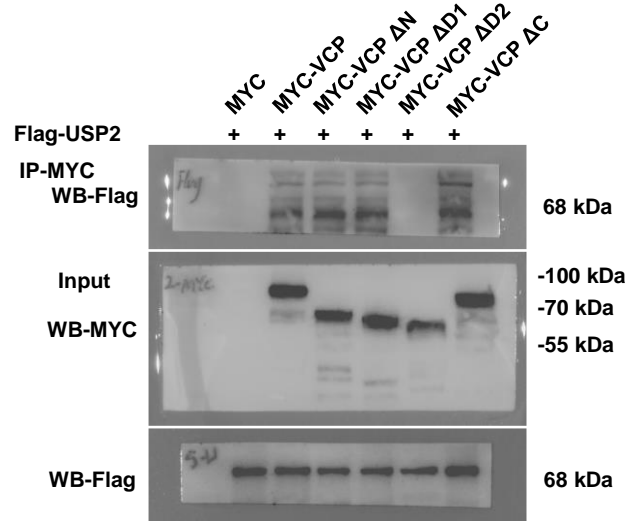

A

Fig. 6

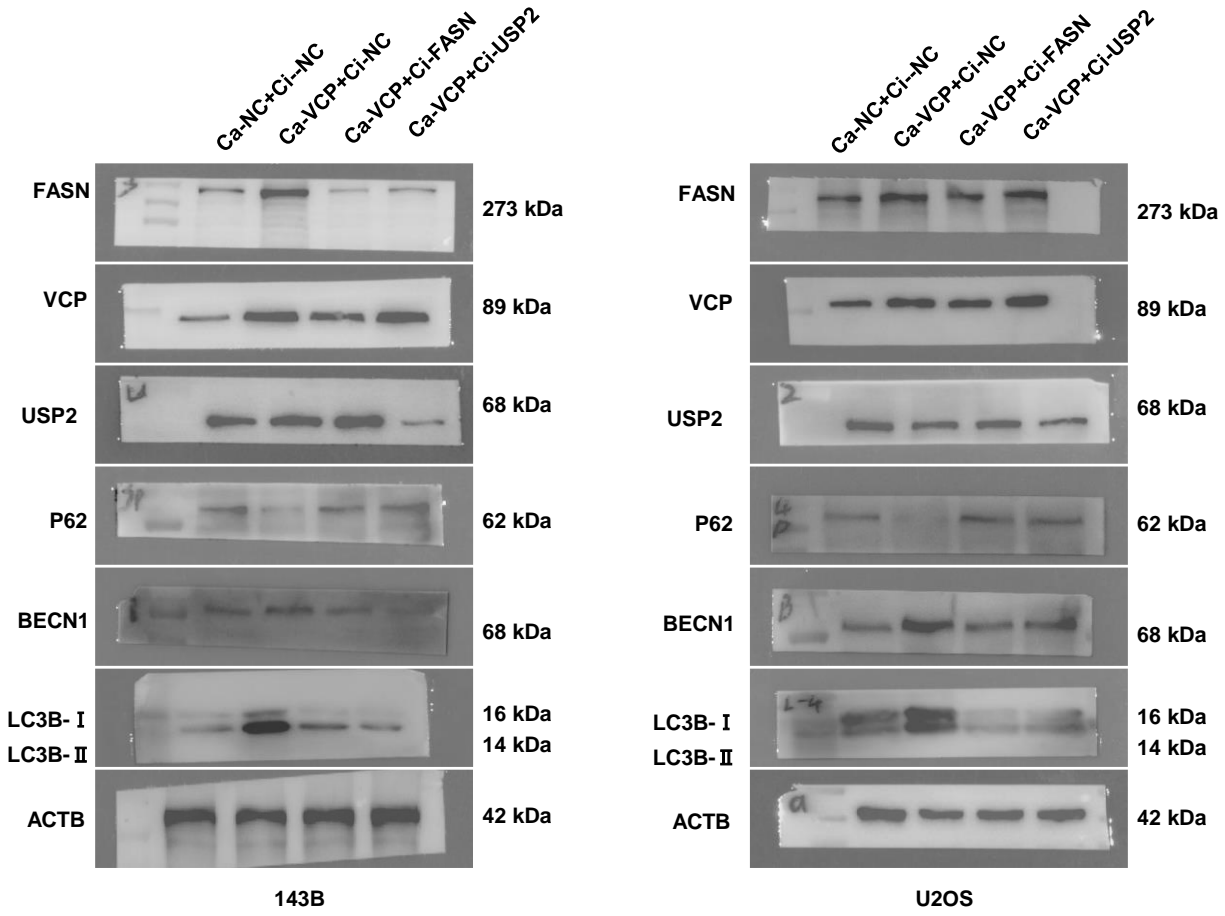

**A**

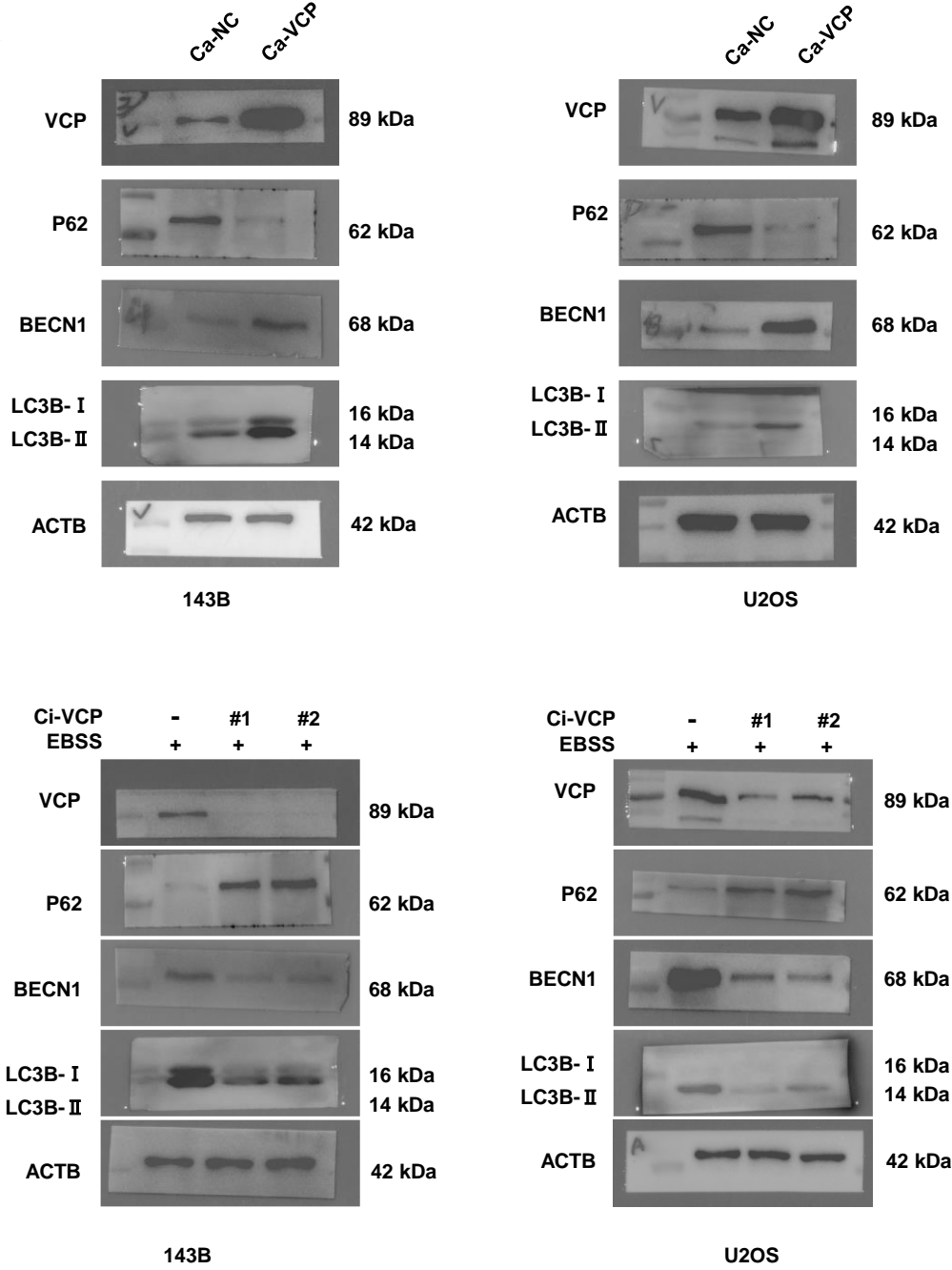

C

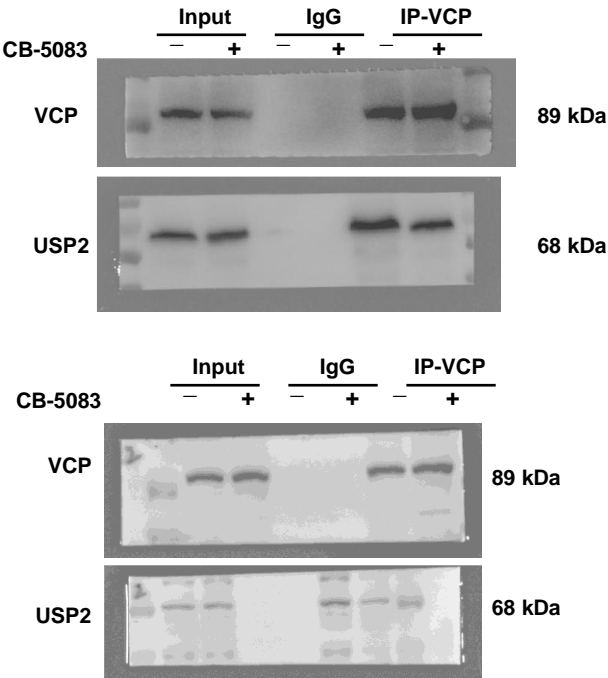

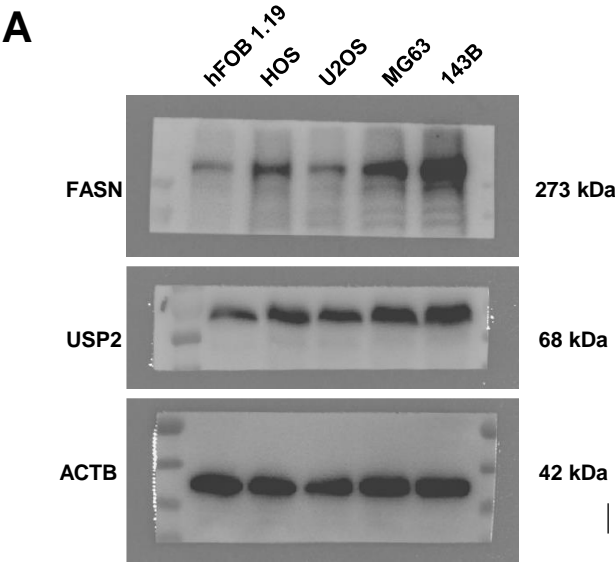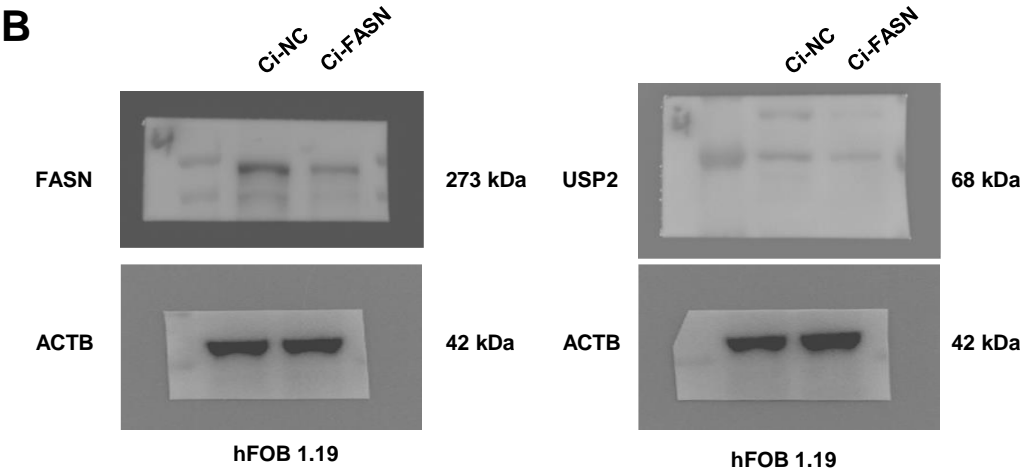

Supplement: Supplementary file 2 — original western blots [file 41419_2024_7168_MOESM2_ESM.pdf]
