# Supplementary material for: VCP enhances autophagy-related osteosarcoma progression by recruiting USP2 to inhibit ubiquitination and degradation of FASN
Source: Cell Death Dis. 2024 Nov 3;15(11):788. doi: 10.1038/s41419-024-07168-6 (PMC11532476; doi:10.1038/s41419-024-07168-6)
Supplement: Supplementary file 3 — Supplementary Tables [file 41419_2024_7168_MOESM3_ESM.docx]

**Table S1 Demonstration of FASN immunoprecipitated proteins.**

| **FASN-IP/MS** | | | | |
| --- | --- | --- | --- | --- |
| FASN  KIF11  PRMT5  WDR77  SLC25A5  IGKV2-29  CLNS1A  PHKB  HSPA8  ACTA1  HSPA1A  RBM10  HRNR  RPS3A  EEF1A1  KCTD5  ALB  TOP1  SF3B3  STK38  SF3B1  TXNDC12  RPS3  TUBB2A  SF3B2  LUC7L2 | \| EIF4B  SNRPD3  TUBA1B  RIOK1  RPS16  PPIB  RPS5  RPS4X  HNRNPA1  SNRPD1  ACTN1  PHKG2  RPS24  RPS2  PHKA2  POLD1  RPS9  MMTAG2  PHF5A  RPS10  RPS8  RPS18  SNRPD2  RPL38  SNRPB  EEF2 \| \| --- \| | SF3A2  EIF3B  RPS15  SUPT16H  U2AF1  RPL27A  HSPA9  ADGRL2  EIF3K  EPS8L2  SUN2  HSPB1  MDK  TECR  HSPA5  TBC1D10B  CPSF6  TIMM50  DNAJA2  SF3B5  TXN  PRPS2  RPLP0  UBR5  UBA52  RPL6 | \| DHX15  RPS13  DDX46  VCP  EIF3CL  PRDX1  RPS7  PRPF38B  CALM3  SNRPA1  KCTD17  RPS19  SLC25A3  EIF3I  PHKA1  APOBEC3C  SNRPE  FAU  CPSF7  KDELR2  RPS20  RPS25  RPS15A  RPS26  ATP1A2  NCL \| \| --- \| | LARP1  RPS27L  EIF3G  USF3  GLO1  IMMT  RPS6  MICOS13  RPL18  XPNPEP3  XAGE1A  RPL13  H2BC21  LSM4  FLG2  RPL3  CCAR1  TPM4  RPS12  RPS11  HNRNPK  RPL21  MBP |

**Table S2 Screening for Autophagy Database.**

| **Autophagy Database** | | | | |
| --- | --- | --- | --- | --- |
| ABCB6  ABL1  ABL2  ABR  ACAP2  ACAP3  ACBD5  ACTA1  ACTA2  ACTB  ACTC1  ACTG1  ACTG2  ACTR2  ACTR3  ACTR3B  AES  AFG3L2  AGFG1  AKT1  AKT1S1  AKT2  AKT3  ALK  ALPI  ALPL  ALPP  ALPPL2  AMBRA1  ANK1  ANK2  ANK3  ANKK1  ANKRD28  ANKRD44  AP1AR  AP1B1  AP1G1  AP1G2  AP1M1  AP1M2  AP1S1  AP1S2  AP1S3  AP2A1  AP2A2  AP2B1  AP2M1  AP2S1  AP3D1  AP3M1  AP3M2  AP4B1  AP4E1  AP4M1  APAF1  ARAF  ARF6  ARFGEF1  ARFGEF2  ARHGAP10  ARHGAP26  ARHGAP42  ARHGEF17  ARHGEF4  ARHGEF9  ARL2  ARNT  ARNT2  ARNTL  ARNTL2  ATF4  ATG10  ATG101  ATG12  ATG13  ATG14  ATG16L1  ATG16L2  ATG2A  ATG2B  ATG3  ATG4A  ATG4B  ATG4C  ATG4D  ATG5  ATG7  ATG9A  ATG9B  ATM  ATR  AURKA  AXL  BAG3  BCL2  BCL2L1  BCR  BDNF  BECN1  BECN2  BLK  BNIP3  BRAF  BRSK1  BRSK2  CALCOCO2  CAMK1  CAMK1D  CAMK1G  CAMK2B  CAMK2D  CAMK2G  CAMKK1  CAMKK2  CAPN1  CAPN10  CAPN11  CAPN12  CAPN13  CAPN14  CAPN2  CAPN3  CAPN5  CAPN6  CAPN7  CAPN8  CAPN9  CAPNS1  CAPNS2  CASP3  CDK1  CDK11A  CDK13  CDK16  CDK17  CDK18  CDK2  CDK5  CDKL1  CDKL4  CDKL5  CDKN1B  CDKN2A  CELF1  CELF3  CELF5  CHUK | CLEC16A  CLOCK  CLTA  CLTB  CLTC  CSNK1A1L  CSNK1D  CSNK1G2  CSNK2A1  CSNK2A2  CSNK2B  CTSB  CTSD  CTSE  CTSL  CTSV  DAPK1  DAPK3  DAW1  DDIT4  DEPTOR  DES  DIRAS3  DNM1  DNM1L  DNM2  DNM3  DNPEP  DRAM2  E2F1  E2F2  EEF1A1  EEF1A2  EI24  EIF2AK1  EIF2AK2  EIF2AK3  EIF2AK4  EIF2S1  EIF4E  EIF4E1B  EIF4EBP1  EIF4EBP2  EIF4EBP3  EPAS1  EPG5  EPHA2  EPHA3  EPHA4  EPHA5  EPHB1  EPHB2  EPHB3  EPN1  EPN2  EPN3  ERBB2  ERBB3  ERBB4  ERN1  ERN2  EVA1A  EXOC1  EXOC2  EXOC3  EXOC3L1  EXOC3L4  EXOC4  EXOC5  EXOC6  EXOC6B  EXOC7  EXOC8  FBXW7  FGFR1  FGFR2  FGFR3  FGR  FIS1  FKBP1A  FKBP8  FOS  FOXO1  FOXO3  FUNDC1  GABARAP  GABARAPL1  GABARAPL2  GABARAPL3  GAPDH  GAPDHS  GBF1  GFAP  GLIPR2  GOPC  GRAMD1B  GRB2  HARBI1  HCK  HDAC1  HDAC4  HDAC5  HDAC6  HDAC7  HIF1A  HIF3A  HRAS  HSPA1A  HSPA1B  HSPA1L  HSPA2  HSPA5  HSPA6  HSPA8  HUNK  IARS  IGF1R  INS  INSR  INSRR  IRGM  IRS1  IRS2  IRS4  ITPR1  ITPR2  ITPR3  JUN  KAT5  KAT6A  KAT6B  KAT7  KAT8  KEAP1  KIAA0226L  KLHL1  KLHL12  KLHL13  KLHL17  KLHL18  KLHL2  KLHL20  KLHL24  KLHL28  KLHL4  KLHL5  KLHL8  KPNA6  KRAS | KRT5  KRT6A  KRT6B  KRT6C  KRT71  KRT72  KRT73  KRT74  KRT75  KRT76  KRT79  KRT8  KRT81  KRT82  KRT83  KRT84  KRT85  KSR1  LAMP1  LAMP2  LATS2  LCK  LPIN1  LPIN2  LRBA  LRSAM1  LYST  MAN2A2  MAN2B1  MAN2C1  MAP1LC3A  MAP1LC3B  MAP1LC3C  MAP2K1  MAP2K2  MAP2K3  MAP2K4  MAP2K5  MAP2K7  MAP3K14  MAP3K6  MAP4K1  MAP4K2  MAP4K3  MAP4K5  MAPK1  MAPK10  MAPK14  MAPK15  MAPK3  MAPK7  MAPK8  MAPK8IP1  MAPKAP1  MARK1  MARK2  MARK3  MARK4  MELK  MERTK  MFN1  MFN2  MINK1  MITF  MLST8  MTM1  MTMR1  MTMR14  MTMR2  MTMR4  MTMR6  MTMR7  MTMR8  MTMR9  MTOR  MTRF1L  MX1  MX2  MYD88  MYLK  MYLK3  MYO3A  NAPSA  NBEA  NBR1  NCOA4  NFE2L1  NFE2L2  NFE2L3  NFKB1  NFKB2  NFKBIA  NFKBIB  NFKBID  NFKBIE  NFKBIZ  NLRC3  NLRC5  NLRP1  NLRP12  NLRP13  NLRP14  NLRP3  NLRP4  NLRP5  NLRP7  NLRP8  NLRP9  NOD1  NOD2  NPAS2  NPAS3  NRAS  NRBF2  NTRK2  NUAK1  NUAK2  NVL  OPA1  OPHN1  PA2G4  PAK1  PAK2  PAK3  PAK4  PAK6  PAK7  PARK2  PDK1  PDPK1  PEX1  PEX5  PEX5L  PGA3  PGA4  PGA5  PGAM5  PI4KB  PIK3C2A  PIK3C2B  PIK3C3  PIK3CA  PIK3CB  PIK3CD  PIK3CG  PIK3R4  PINK1  PIP4K2A  PIP5K1A | \| PIP5K1B  PIP5K1C  PKN1  PKN2  PKN3  PLCB4  PLCD1  PLCG1  PLCH1  PLCH2  PLD1  PLD2  PNCK  POC1A  POC1B  PPP1CA  PPP1CC  PPP2CA  PPP2CB  PPP2R1A  PPP2R1B  PPP2R2A  PPP2R2B  PPP2R2C  PPP2R2D  PPP2R3A  PPP2R3B  PPP2R3C  PPP2R4  PPP2R5A  PPP2R5B  PPP2R5C  PPP2R5D  PPP2R5E  PPP4C  PPP6C  PREX1  PREX2  PRKAA1  PRKAA2  PRKAB1  PRKAB2  PRKACA  PRKACB  PRKCA  PRKCB  PRKCD  PRKCE  PRKCG  PRKCH  PRKCI  PRKCQ  PRKCZ  PRKDC  PRMT1  PRMT3  PRMT8  PRR5  PRR5L  PTEN  RAB10  RAB12  RAB1A  RAB1B  RAB33B  RAB5A  RAB5B  RAB5C  RAB7A  RAB8A  RAB9A  RABGEF1  RAF1  RALA  RALB  RALGAPA1  RALGAPA2  RB1CC1  RCAN1  REL  RELA  RELB  RET  RGL1  RGS19  RHEB  RICTOR  RIPK1  RIPK4  RPS6KA1  RPS6KA3  RPS6KA4  RPS6KA6  RPS6KB1  RPS6KB2  RPTOR  RRAGA  RRAGB  RRAGC  RRAGD  RUBCN  RUNX1  RUNX3  RYR1  RYR2  SAR1A  SAR1B  SBF2  SCOC  SEC16A  SEC16B  SEC23A  SEC23B  SEC24B  SEC24C  SEC24D  SESN1  SESN2  SESN3  SGK2  SH3GL1  SH3GL2  SH3GL3  SH3GLB1  SH3GLB2  SHC1  SHC2  SHC3  SHC4  SIDT1  SIDT2  SIK1  SIK2  SIK3  SIM2  SIN3A  SIN3B  SIPA1L3  SIRT1  SLC1A1  SLC1A2  SLC1A4  SLC1A5  SLC1A6  SLC1A7  SLC36A1  SLC36A4  SLC3A1  SLC3A2 \| \| --- \| | SLC7A10  SLC7A11  SLC7A5  SLC7A6  SLC7A7  SLC7A8  SLC7A9  SMPD1  SMPDL3B  SNAP29  SNRK  SNRNP40  SNX1  SNX30  SNX4  SOD1  SOD2  SOS1  SOS2  SPATA13  SPATA5  SPATA5L1  SPG7  SQSTM1  SRC  STK11  STK24  STK25  STK26  STK36  STK38L  STK4  STX17  STX7  STX8  STXBP1  STXBP2  STXBP3  SUPT20H  TBC1D12  TBC1D14  TBC1D25  TBC1D4  TBC1D5  TBC1D7  TBK1  TECPR1  TELO2  TEP1  TFE3  TFEB  TGFBRAP1  TIGAR  TM9SF1  TM9SF2  TM9SF3  TM9SF4  TMEM173  TMEM74  TNFAIP2  TNIK  TNS1  TNS2  TNS3  TOMM7  TOR1B  TP53INP2  TPR  TPTE2  TRADD  TRAF1  TRAF2  TRAF3  TRAF4  TRAF5  TRAF6  TRAPPC1  TRAPPC10  TRAPPC2  TRAPPC3  TRAPPC4  TRAPPC5  TRAPPC6A  TRAPPC6B  TRAPPC8  TRAPPC9  TRRAP  TSC1  TSC2  UBB  UBC  UBE2A  UBE2D2  UBE2D3  UBE2H  UBE2J2  UBE2L3  UBE2N  UGT1A1  UGT1A10  UGT1A6  UGT1A9  UGT2A1  UGT2A3  UGT2B10  UGT2B15  UGT2B17  UGT2B28  UGT2B4  UGT2B7  ULK1  ULK2  ULK3  UVRAG  VAMP3  VAMP7  VAMP8  VCP  VDAC1  VDAC2  VDAC3  VIM  VMP1  VPS13A  VPS13C  VPS16  VPS18  VPS33A  VPS33B  VPS39  VPS41  VPS45  VTI1B  WAC  WDFY3  WDFY4  WDR3  WDR45  WDR45B  WDR5  WDR5B  WIPI1  WIPI2  YES1  YME1L1  ZFYVE1 |

**Table S3 Demonstration of VCP immunoprecipitated proteins.**

| **VCP IP/MS (Top 200)** | | | | |
| --- | --- | --- | --- | --- |
| \| MYH9 \| \| --- \| \| ACTB \| \| PLEC \| \| MYH10 \| \| VIM \| \| SPTBN1 \| \| SPTAN1 \| \| MYO6 \| \| RAI14 \| \| MYO1C \| \| MPRIP \| \| MYO1B \| \| MYO5A \| \| DBN1 \| \| DYNC1H1 \| \| LIMA1 \| \| MYL6 \| \| H2AC14 \| \| FLNB \| \| SVIL \| \| MYO1E \| \| MYL12A \| \| AHNAK \| \| CLTC \| \| ITPR3 \| \| TUBB \| \| FLNA \| \| SPECC1L \| \| ACTN1 \| \| H4C1 \| \| KRT18 \| \| PARP1 \| \| FLII \| \| TUBA1B \| \| PDCD11 \| \| H2BC13 \| \| PPP1R12A \| \| DHX9 \| \| LMO7 \| \| HSPA8 \| | \| EEF1A1 \| \| --- \| \| MYL6B \| \| TOP1 \| \| DDX21 \| \| MACF1 \| \| NPM1 \| \| TPM3 \| \| MYO18A \| \| MYBBP1A \| \| SFPQ \| \| PRPF8 \| \| TMPO \| \| RPL10A \| \| PRKDC \| \| HNRNPM \| \| RPS4X \| \| ANXA2 \| \| COPA \| \| DHX30 \| \| LUZP1 \| \| DDX17 \| \| PHGDH \| \| RPS3 \| \| RPL3 \| \| RPL5 \| \| NOP56 \| \| ATP5F1B \| \| HSP90AB1 \| \| RPL7 \| \| RPL4 \| \| SNRNP200 \| \| PKM \| \| HSPA9 \| \| TUFM \| \| NONO \| \| TJP2 \| \| NCL \| \| TMOD3 \| \| GAPDH \| \| HNRNPH1 \| | \| RPL7A \| \| --- \| \| HNRNPU \| \| DOCK7 \| \| GSN \| \| EEF2 \| \| DDX5 \| \| FLNC \| \| UACA \| \| SUPT16H \| \| DHX15 \| \| HSPD1 \| \| ACTR3 \| \| RPLP0 \| \| HSPA5 \| \| ATP5F1A \| \| CAD \| \| FXR1 \| \| ACTR2 \| \| RPS3A \| \| TJP1 \| \| SMARCA5 \| \| FRYL \| \| PABPC1 \| \| IMPDH2 \| \| IMMT \| \| LRRFIP2 \| \| ARPC2 \| \| RPS2 \| \| ITPRID2 \| \| RPL13A \| \| SSRP1 \| \| SF3B3 \| \| PPP1R9B \| \| NOP58 \| \| SYNCRIP \| \| MATR3 \| \| AP2B1 \| \| CKAP4 \| \| LARP1 \| \| SERBP1 \| | \| RPL17 \| \| --- \| \| FBL \| \| LRPPRC \| \| IQGAP1 \| \| HNRNPK \| \| TUBB6 \| \| ATAD3A \| \| HNRNPC \| \| HNRNPL \| \| RPL10 \| \| EIF4A1 \| \| PPP1CB \| \| RPS11 \| \| CEBPZ \| \| G3BP1 \| \| NUMA1 \| \| DDX1 \| \| CORO2B \| \| ANPEP \| \| RPS9 \| \| MAP1B \| \| VCP \| \| RPS15A \| \| ILF3 \| \| PUM3 \| \| LRRFIP1 \| \| BRIX1 \| \| NOL6 \| \| SF3B1 \| \| MYO1D \| \| RPLP2 \| \| LMNB1 \| \| HNRNPA2B1 \| \| KHDRBS1 \| \| DDX24 \| \| CORO1C \| \| RUVBL1 \| \| RTCB \| \| IGF2BP3 \| \| DDX3X \| | \| ARPC1B \| \| --- \| \| SIPA1L1 \| \| DKC1 \| \| STAU1 \| \| LMNA \| \| RPL9 \| \| RPL8 \| \| RPL6 \| \| RRP1B \| \| EFTUD2 \| \| GNL3 \| \| NAT10 \| \| RUVBL2 \| \| ACTN4 \| \| MACROH2A1 \| \| RSL1D1 \| \| AP2A1 \| \| ENO1 \| \| TPM1 \| \| HNRNPA1 \| \| FUS \| \| NOP2 \| \| CAPZA1 \| \| RPL23A \| \| RPL23 \| \| TUBB4B \| \| H3C1 \| \| PFKP \| \| CHD4 \| \| KPNB1 \| \| PCM1 \| \| MICAL3 \| \| RRBP1 \| \| EIF2S1 \| \| ARF4 \| \| USP2 \| \| FXR2 \| \| HNRNPF \| \| SMTN \| \| RPS14 \| |

**Table S4 List of related deubiquitylation-related proteins from Epithelial Systems Biology Laboratory.**

| **deubiquitylation-related proteins** | | | | |
| --- | --- | --- | --- | --- |
| \| ATXN3 \| \| --- \| \| ATXN3L \| \| BAP1 \| \| BRCC3 \| \| COPS5 \| \| COPS6 \| \| CYLD \| \| DESI1 \| \| DESI2 \| \| EIF3F \| \| EIF3H \| \| JOSD1 \| \| JOSD2 \| \| MPND \| \| MYSM1 \| \| OTUB1 \| \| OTUB2 \| \| OTUD1 \| \| OTUD3 \| \| OTUD4 \| \| OTUD5 \| \| OTUD6A \| \| OTUD6B \| \| OTUD7A \| | \| OTUD7B \| \| --- \| \| OTULIN \| \| PAN2 \| \| PARP11 \| \| PRPF8 \| \| PSMD14 \| \| PSMD7 \| \| SENP1 \| \| SENP2 \| \| SENP3 \| \| SENP5 \| \| SENP6 \| \| SENP7 \| \| SENP8 \| \| STAMBP \| \| STAMBPL1 \| \| UCHL1 \| \| UCHL3 \| \| Uchl4 \| \| UCHL5 \| \| USP1 \| \| USP10 \| \| USP11 \| \| USP12 \| | \| USP13 \| \| --- \| \| USP14 \| \| USP15 \| \| USP16 \| \| USP17L10 \| \| USP17L11 \| \| USP17L12 \| \| USP17L13 \| \| USP17L15 \| \| USP17L17 \| \| USP17L18 \| \| USP17L19 \| \| USP17L20 \| \| USP17L21 \| \| USP17L22 \| \| USP17L3 \| \| USP17L4 \| \| USP17L5 \| \| USP17L7 \| \| USP17L8 \| \| USP17La \| \| Usp17lb \| \| Usp17lc \| \| Usp17ld \| | \| USP17Le \| \| --- \| \| USP18 \| \| USP19 \| \| USP2 \| \| USP20 \| \| USP21 \| \| USP22 \| \| USP24 \| \| USP25 \| \| USP26 \| \| USP27X \| \| USP28 \| \| USP29 \| \| USP3 \| \| USP30 \| \| USP31 \| \| USP32 \| \| USP33 \| \| USP34 \| \| USP35 \| \| USP36 \| \| USP37 \| \| USP38 \| \| USP39 \| | \| USP4 \| \| --- \| \| USP40 \| \| USP41 \| \| USP42 \| \| USP43 \| \| USP44 \| \| USP45 \| \| USP46 \| \| USP47 \| \| USP48 \| \| USP49 \| \| USP5 \| \| USP50 \| \| USP51 \| \| USP53 \| \| USP54 \| \| USP6NL \| \| USP7 \| \| USP8 \| \| USP9X \| \| USP9Y \| \| USPL1 \| \| USPL1 \| \| VCPIP1 \| \| YOD1 \| \| ZRANB1 \| |

**Table S5 Primer sets used for qPCR, RT-PCR.**

| **Primer set 1** | **Primers** | **Sequence** | **Product size(bp)** | |
| --- | --- | --- | --- | --- |
| ACTB | Forward  Reverse | 5′-TGCCCATCTACGAGGGGTATG-3′  5′-TCTCCTTAATGTCACGCACGATTT-3′ | | 156 |
| FASN | Forward  Reverse | 5′-CAACTCACGCTCCGGAAA-3′  5′-TGTGGATGCTGTCAAGGG-3′ | 310 | |
| VCP | Forward  Reverse | 5′- GCAGTTGTTCCGAGGTGACA-3′  5′- GCACATGGATACGTTTGCCG-3′ | 203 | |

**Table S6 Oligonucleotide sets used for constructs.**

| **Oligo set** | **Sequence** |
| --- | --- |
| CRISPRi-FASN  sgRNA-FASN-1 Target  sgRNA-FASN-2 Target | 5′- GGCCGCGGTTTAAATAGCGTCGG -3′  5′- GGTTTAAATAGCGTCGGCGCCGG -3′ |
| CRISPRi-VCP  sgRNA-VCP-1 Target  sgRNA-VCP-2 Target | 5′- TTCGCCCGACGCCTCGCTGCCGG -3′  5′- AGCGAGAGGGAAGCCGCTTGCGG -3′ |
| CRISPRi-USP2  sgRNA-USP2-1 Target  sgRNA-USP2-2 Target  CRISPRa-FASN  sgRNA-FASN-1 Target  sgRNA-FASN-2 Target  CRISPRa-VCP  sgRNA-VCP-1 Target  sgRNA-VCP-2 Target | 5′- ACTCAGTGACGCGAGACGCGGGG -3′  5′- CGTCATTAGAGCCCGGCCTCCGG -3′  5′- ATTTAAACCGCGGCCATCCCCGG -3′  5′- AGCGTCGGCGCCGGCCTAGAGGG -3′  5′- AGCGTTGCGGCCAATTGACGTGG -3′  5′- GGCGTGTCGCATCACTGAGGCGG -3′ |
